# Three genetically distinct ferlaviruses have varying effects on infected corn snakes (*Pantherophis guttatus*)

**DOI:** 10.1371/journal.pone.0217164

**Published:** 2019-06-04

**Authors:** Michael Pees, Volker Schmidt, Tibor Papp, Ákos Gellért, Maha Abbas, J. Matthias Starck, Annkatrin Neul, Rachel E. Marschang

**Affiliations:** 1 Department for Birds and Reptiles, University Teaching Hospital, University of Leipzig, Leipzig, Germany; 2 Institute for Veterinary Medical Research, Centre for Agricultural Research, the Hungarian Academy of Sciences, Budapest, Hungary; 3 Institute for Environmental and Animal Hygiene, University of Hohenheim, Stuttgart, Germany; 4 Department of Biology II LMU München–Biocenter, Munich, Germany; 5 Laboklin, Bad Kissingen, Germany; University of Valencia, SPAIN

## Abstract

Ferlaviruses are important pathogens in snakes and other reptiles. They cause respiratory and neurological disease in infected animals and can cause severe disease outbreaks. Isolates from this genus can be divided into four genogroups–A, B, and C, as well as a more distantly related sister group, “tortoise”. Sequences from large portions (5.3 kb) of the genomes of a variety of ferlavirus isolates from genogroups A, B, and C, including the genes coding the surface glycoproteins F and HN as well as the L protein were determined and compared. *In silico* analyses of the glycoproteins of genogroup A, B, and C isolates were carried out. Three isolates representing these three genogroups were used in transmission studies with corn snakes (*Pantherophis guttatus*), and clinical signs, gross and histopathology, electronmicroscopic changes in the lungs, and isolation of bacteria from the lungs were evaluated. Analysis of the sequences supported the previous categorization of ferlaviruses into four genogroups, and criteria for definition of ferlavirus genogroups and species were established based on sequence identities (80% resp. 90%). Analysis of the ferlavirus glycoprotein models showed parallels to corresponding regions of other paramyxoviruses. The transmission studies showed clear differences in the pathogenicities of the three virus isolates used. The genogroup B isolate was the most and the group A virus the least pathogenic. Reasons for these differences were not clear based on the differences in the putative structures of their respective glycoproteins, although e.g. residue and consequential structure variation of an extended cleavage site or changes in electrostatic charges at enzyme binding sites could play a role. The presence of bacteria in the lungs of the infected animals also clearly corresponded to increased pathogenicity. This study contributes to knowledge about the structure and phylogeny of ferlaviruses and lucidly demonstrates differences in pathogenicity between strains of different genogroups.

## Introduction

The first description of a ferlavirus outbreak was documented in Switzerland in 1972 [1]. Since then, ferlavirus infections have been found in different parts of the world, in various reptile species, and with varying clinical signs [2]. The virus associated with that initial outbreak was named Fer-de-Lance Virus (FDLV) [3], and then described as ophidian paramyxovirus (PMV). Today this and related viruses are classified in the genus *Ferlavirus* in the family *Paramyxoviridae* [4]. Ferlaviruses have been detected in a variety of snake species, including elapids, colubrids, crotalids, boids and pythonids [5,6]. In snakes, ferlavirus infections can lead to severe clinical disease, and can cause devastating outbreaks in both private and zoological collections [5]. Clinical signs mainly involve the respiratory and central nervous system. Anorexia, emaciation and sudden death are also common. The severity of the signs found varies, depending on the individual outbreak. In general, viperid species seem to be especially susceptible to disease, but other snakes can also be affected severely [2,5]. The outcome is often fatal once clinical signs develop.

Besides snakes, lizards [7–11] and tortoise species [11,12] can be infected. Clinical signs in these animals can parallel those described in viperid snakes, especially pneumonia, but disease is observed more sporadically, and virus has been detected in apparently clinically healthy lizards in some cases.

Formal pathogenesis of ferlavirus induced pneumonia was described in experimentally infected vipers, thereby fulfilling Koch’s postulates [13]. Five Aruba island rattlesnakes (*Crotalus durissus unicolor)* were inoculated intratracheally with a ferlavirus cell culture isolate, and pulmonary lesions were found in infected snakes within only a few days. All snakes developed severe interstitial pulmonary disease, including proliferation and vacuolation of the faveolar epithelial cells. Those animals which were not euthanized died between days 19 and 22 after infection. Cuboidal metaplasia and hypertrophy of the type I respiratory epithelial cells and hypertrophy of the type II respiratory epithelial cells, an increased secretory activity of type II cells, desquamation of respiratory epithelial cells, invasion by bacterial pathogens, infiltration of the interstitium with heterophils as well as lymphocytes, histiocytes and plasma cells, oedema, fibrin deposition and, depending on the stage of the inflammation, fibrous metaplasia of the lung tissue, are common findings [5,13,14]. Intracytoplasmic inclusion bodies have been described in affected epithelial cells in the lung, but are reported to be uncommon [5,14].

A study in Burmese pythons (*Python molurus*) compared the histological and ultrastructural lung morphology between healthy snakes and snakes suffering from respiratory diseases. The authors demonstrated a massively thickened air-blood barrier and reduction of the lung exchange capacity in the majority of snakes that tested positive for ferlaviruses by virus isolation and/or PCR. Hyperplasia of the ciliated cells and type II pneumocytes was found, and the epithelium appeared pseudostratified to multilayered, overgrowing the faveolar capillaries [15].

In addition to the lungs, the central nervous system (CNS) is known to be a target organ for ferlavirus infections, causing nonsuppurative meningoencephalitis [16] or demyelinisation and degeneration of axon sheets [17]. Another organ that is reported to be possibly affected is the pancreas, resulting in pancreatitis and/or pancreas necrosis [5].

It is suspected that the course of the pathological alterations might also be influenced by Gram-negative bacteria, especially *Salmonella* spp., which are often found in affected lung tissue [5,18]. Further, concurrent virus infections (reovirus, adenovirus) and even dual infections with two different ferlavirus strains have been detected in snakes that exhibited a broad range of clinical signs, and the authors discussed that this might also have an influence on the pathogenesis of the infection [19].

A number of publications have compared various ferlavirus isolates, based mostly on partial L, F, and HN gene sequences [11,12,19–25]. These studies have shown that the genus can be divided into four different genogroups. Viruses belonging to genogroups A and B have been found in squamates and chelonians, whereas viruses of genogroup C have been found in squamates only, so far. The fourth group has been called „tortoise”and is currently only represented by a single virus isolate, found in a Hermann’s tortoise (*Testudo hermanni*) with pneumonia [11,12,19,24,25].

All PMVs contain two large glycosylated envelope proteins: the attachment protein (HN/H/G) and the fusion protein (F) which in cooperation with each other orchestrate viral entry into the cells [26,27]. The attachment protein, which is considered the most important antigenic determinant of paramyxoviruses [28], is a homotetrameric type II integral membrane protein, and is responsible for binding to the cell receptor. The attachment protein has four conserved domains starting from the amino terminus: a cytoplasmatic tail, a hydrophobic transmembrane region, a stalk domain and a globular head.

The other major envelope component, the fusion protein (F), is a homotrimeric type I membrane glycoprotein, and it directly mediates membrane fusion [29]. Typical for PMVs is that the F protein is synthesized as an inactive precursor F0, which requires proteolytic cleavage into disulfide-linked F1+F2 heterodimers via cellular proteases that are provided by the host [30]. Cleavage of the F protein is an essential step in the pathogenic process, and in ferlaviruses, like in many other PMVs, it is promoted by a ubiquitous endoprotease, furin, during transport through the trans-Golgi network [22]. As described a large number of times, this enzyme recognizes the R-X-K/R-R motif and cuts at the C-terminus of the basic residues of this cleavage site (CS). A much broader effective recognition site has also been ascertained for furin in several studies [31,32]. Further important domains in the F protein are the signal peptide (SP) at the N-terminal, the trans-membrane (TM), and the cytoplasmatic tail (CT) domain at the C-terminal.

The F proteins of several avian and mammalian PMVs have been demonstrated to be key determinants of viral virulence and structural changes and/or inhibitions may alter the pathogenic character of the bearer virus [29]. Partial F gene sequence of an FDLV strain was first published following accidental detection among expressed sequence Tags (ESTs) generated from venom glands of wild caught Fer de Lance snakes [33]. Papers comparing and grouping more than a dozen ferlavirus isolates based on partially sequenced F, HN and L genes were also published representing the groups A and B of today’s classification [20–23]. In the latter, the complete F gene of an isolate from a red-tailed green ratsnake (*Gonyosoma oxycephalum*; GonoGER85) was compared with its homolog in FDLV [22], both members of group A [11]. This study also included expression and *in vitro* functional analysis of certain F protein domains and described the CS, FP, HRA and HRB-LZ motifs. In this report HR1 and HR2 were referred to as HRA and HRB, respectively. In addition to FDLV [34], full genome sequences are available from five almost identical anaconda ferlaviruses from a severe disease outbreak in Hong-Kong [35] as well as from a Chinese watersnake (*Myrrophis chinensis*) (GenBank Acc.No: MG600060, group B), and from captive rattlesnakes (*Crotalus durissus terrificus*) in Brazil (MH411104, group A) (both unpublished). Thus, complete F and HN protein sequences of both group A and B members have been made available, yet so far no comparative structural analysis of these has been performed.

Although the reports mentioned earlier show that clinical signs of disease may vary depending on the virus strain as well as the snake species affected, little is known about possible differences in the virulence between virus strains in the same snake species and under similar or identical conditions. Based on their importance for entry into host cells and their role in virus replication and egress from host cells, we hypothesized that the F and HN glycoproteins could influence the pathogenicity of ferlaviruses. It was therefore the aim of this study to comparatively examine the pathogenesis of three ferlavirus strains belonging to genogroups A, B, and C in a model species under experimental standardized conditions, and to genetically characterize and *in silico* analyze their surface glycoproteins. The infection studies on which this data is based have been described previously [15,36,37] regarding virus detection in the infected snakes. The present report details the pathological, histological, and bacteriological findings in the infected snakes as well as the partial molecular characterization of the viruses used in the study.

## Materials and methods

### Virus isolates for RT-PCR amplification, sequencing and phylogenetic analysis

Nine available squamate ferlavirus isolates (see S1 Table) from genogroups A, B and C, most of which had been earlier characterized based on partial U, HN and L gene sequences [11,25], were used for RT-PCRs and sequencing, in order to determine their complete F and HN genes. All isolates were propagated in viper heart cells (VH2) as described previously [25]. RNA was prepared from 300 μl of cell culture supernatant of each isolate using the guanidinium isothiocyanate method as described previously [38] and resuspended in 75 μl of RNase free water. RT-PCRs targeting a portion of the L gene and a portion of the HN gene of ophidian PMVs were carried out as described by Ahne et al. [20]. The primary RT-PCR targeting the F-gene was performed as discribed by Franke and coworkers [21]. Additional RT-PCRs were performed as described previously [11,25]. Briefly, the reactions were carried out in 25 μl reaction mixtures containing 2.5 μl of prepared RNA, 1 μM of each primer, 1 x Taq buffer with KCl, 2.5 mM MgCl_2_, 0.2 mM of each dNTP, 70 units reverse transcriptase, 6 units ribonuclease inhibitor, and 1.25 units Taq polymerase (all Fermentas, St Leon-Rot, Germany). In the second rounds of the nested PCRs, 1μl of the first round reaction, 1μM of each primer, 1× Taq buffer with KCl, 2.5mM MgCl2, 0.2mM of each dNTP, and 1.25 units Taq were included in 25μl reaction mixtures. The thermal cycling protocol described by Ahne et al. [20] was taken as a basis and was adjusted taking into account the T_m_ of the applied primers and the length of the expected PCR amplicon.

Complete F gene sequences from GenBank (FDLV, GenBank Accession No: AY141760; Gono-GER85, AY725422) and a partial F gene sequence from the tortoise isolate THER GER99 ([11], unpublished sequence) were aligned and used to design new consensus primers (S2 Table). Various primer combinations were applied in order to obtain F and HN sequences from each isolate (S3 Table). All RT-PCR products were separated by gel electrophoresis and visualized by ethidium bromide staining under UV light (320 nm). Amplicons were purified by gel extraction (Invisorb Spin DNA Extraction Kit; Invitek GmbH, Berlin, Germany) and Sanger sequencing was carried out directly by a commercial service (Eurofins MWG Operon, Ebersberg, Germany). Each PCR product was sequenced at least twice, questionable sequences were confirmed by additional PCRs and sequencing.

ABI automated sequencer generated reads were edited, corrected, and joined using Staden-Package version 2003.0 Pregap4 and Gap4 programs as described [39]. Edited sequences were compared to GenBank databases http://blast.ncbi.nlm.nih.gov/Blast.cgi, using BLASTN and BLASTX algorithms. Alignments and similarity matrices were generated using the BioEdit Sequence Alignment Editor programme [40] and/or the Geneious 9.1.8 program (https://www.geneious.com). Nucleotide sequences of the obtained genome segments and those of the homologous parts of the ferlaviruses available in GenBank as well as other PMVs, were aligned using the MAFFT (G-INS-i) algorithm of the Geneious programme [41]. Obtained nucleotide alignments were edited using a GBlocks Server (http://molevol.cmima.csic.es/castresana/Gblocks_server.html) or manually optimizing for a better phylogenetic resolution among ferlaviruses. Phylogenetic molecular evolutionary analyses were conducted using MEGA version 10.0.2 [42], Topali v2.5 [43], RAxML-NG v0.7.0 [44] and Phylip v.3.64 [45] programs for model selection, construction of Bayesian and Maximum Likelihood (ML) trees, and for calculating the distance based trees, respectively. For the Bayesian and ML calculations, the general time reversible substitution models with gamma distribution and invariable sites (GTR+G+I) were selected. Two runs of 1 million generations, with a sample frequency of 10 and a burn in ratio of 40% was applied for MrBayes calculations, while 100 and 1000 bootstrap runs with optimized topology and branch lengths were used for PhyML and RaxML calculations, respectively. Another 100 bootstrap runs with the DNAdist program using the Kimura-2 parameter followed by the Fitch-Margoliash algorithm were carried out to construct the distance-based tree. Sequence Demarcation Tool (SDT) programme v.1.2. was used to approximate suitable group demarcation criteria [46]. Pairwise-alignment-based matrix calculations were performed with the SDT programme, based on the nucleotide and amino acid data. A recombination analysis of the unedited nucleotide alignment was performed using the SimPlot for Windows v.3.5.1 programme. Default settings were applied both in the SimPlot (window size: 200, step size: 20, Kimura 2-parameter) and the BootScan (window size: 200, step size: 20, Kimura 2-parameter, NJ tree) analyses.

### Molecular protein analysis, modeling and graphics

A primary protein analysis was carried out using NetNGlyc 1.0 to predict the glycosylation sites, SignalP 4.1 Server, TMHMM Server v. 2.0 to predict the signal peptide and transmembrane motifs (both from the Center for Biological Sequence Analysis, Denmark), and GENtle, which was used to predict hydrophobicity plots according to Kyte and Doolittle [47]. Secondary structures and transmembrane domains were also visualized using the SABLE server (http://sable.cchmc.org/) and the Phobious (http://phobius.sbc.su.se/) combined transmembrane topology and signal peptide predictor was also tested on the data, as well as the InterProScan site of the EMBL-EBI (https://www.ebi.ac.uk/interpro/sequencesearch/iprscan5).

F and HN proteins of three representative squamate ferlavirus group members (A: Xeno-USA99, B: Crot-GER03 and C: PanGut-GER09, the ones also included in the animal trials) were modeled using the Phyre2 [48] and the I-TASSER [49] automatic protein structure modeling servers. Supplementary table (S4 Table) contains the detailed statistical evaluation of the generated models. We used PROCHECK [50] to calculate the Ramachandran plots and two independent protein structure quality evaluation methods, PROSESS [51] and ProSA [52], were used to characterize the models. Phyre2 generates only one output model using several templates, while I-TASSER creates five models using fewer templates which are ranked by confidence scores (C-score). Only I-TASSER models with the best C-score were used in this study. The protein model evaluation statistics show that Phyre2 generated slightly better models for F proteins while I-TASSER created slightly better models for HN.

The N-terminal (amino acids (aa) 1–20) and C-terminal (aa 483–545) regions were deleted from the final F protein models because these regions had very low confidence values. The trimer F proteins were built with the academic version of the Schrödinger Maestro (https://www.schrodinger.com/freemaestro [53]), using the biological assembly coordinates of the Nipah virus fusion glycoprotein crystal structure (PDB ID: 5EVM).

For HN proteins, the first 86 residues were omitted from the model structure because of low reliability. The ferlavirus HN dimers were created with the above mentioned Schrödinger Maestro using the A and B chain coordinates of the parainfluenza virus 5 HN which is one of its templates.

All F protein trimer and HN protein dimer structures were refined with UCSF Chimera 1.12. The fifty steepest descent energy minimization steps were applied to eliminate the steric conflicts between the side-chain atoms. The NetPhos 3.1 server (http://www.cbs.dtu.dk/services/NetPhos/, [54]) was used to predict serine, threonine and tyrosine phosphorylation sites in all modelled ferlavirus proteins.

Electrostatic potential maps were calculated with Adaptive Poisson–Boltzmann Solver (APBS) version 1.3 [55] using the linearized Poisson–Boltzmann method [56] with a dielectric constant of 78.0 and 2 for the water solvent and protein core, respectively. The partial charges for the electrostatic potential calculations were calculated with PDB2PQR [57]. Molecular graphics were created with VMD version 1.9.3 [58] and with PyMOL 1.8.4.0 Molecular Graphics System (https://pymol.org/2).

### Animal and virus isolate selection and treatment for the infection study

The experimental setup and the animals used in this study have already been described for the comparison of the ferlavirus detection rates using different methods [36]. A total of 42 adult corn snakes (*Pantherophis guttatus*) were acquired from a commercial company and underwent a thorough health check, including assessment of the clinical status, and examination for ecto- and endoparasites (visual inspection,fecal and cloacal wash sample for native assessment and flotation for parasite eggs) as well as bacterial and fungal pathogens (swabs from choana, cloaca and tracheal wash sample, aerobic culture according to the procedure described below for the lung swabs). Furthermore, a combined tracheal wash and cloacal swab sample was checked for the presence of ferlaviruses following an established protocol [19]. Only unremarkable snakes were included in the study and sorted randomly into three trial groups with 12 snakes each, as well as one untreated control group (six snakes). The snakes were housed under standardized conditions (terraria size approx. 140x78x65 cm, six animals each, temperature 20–32°C with hotspots at 35°C, relative humidity 40–70%, ground material turf, water basin, hiding places). For each group the trial consisted of an acclimatization period of six days, followed by virus (or placebo) application and a 49 days post infection period.

Three different strains were selected for the experimental infection, belonging to the three ferlavirus (geno)groups (A, B and C) described in squamates:

1. Genogroup A: isolate “Xeno-USA99”. This isolate was found in a flathead knob-scaled lizard (*Xenosaurus platyceps*) [11]. The tested lizards were imported from Mexico to the United States and kept in captivity with no contact with other reptiles. It showed no specific clinical signs and remained healthy after testing. The isolate was obtained from a cloacal swab during routine examination.
2. Genogroup B: isolate “Crot-GER03”. This virus strain was isolated from a timber rattlesnake (*Crotalus horridus*) after a fatal outbreak with severe respiratory disease in a mixed collection of various vipers in Germany [11]. Several animals in this collection died. Clinical signs observed included respiratory distress and CNS signs.
3. Genogroup C: isolate “PanGut-GER09”. This isolate was found in a corn snake (*Pantherophis guttatus*) during a disease outbreak with several sudden deaths in a corn snake collection in Germany [25].

All isolates were grown in VH2 according to an established protocol [25]. The total number of passages for the virus strains used prior to inoculation was 9 (Xeno-USA99, PanGut-GER09) and 11 (Crot-GER09). Virus suspensions were concentrated to at least 10^6^ TCID_50_ per ml and stored at -80°C before application. Inoculation was done via the trachea using 1 ml of the thawed suspension (control group 1 ml VH2-cell culture supernatant). After application, the virus concentration of the suspension was rechecked and confirmed to be 10^6.5^ TCID_50_ per ml in all three samples (details see Pees et al. [36]). In this manuscript, we refer to trial group A, B, C according to the viruses used (genogroup A, B, C).

### Animal sampling and processing of samples

After experimental infection, *intra vitam* sampling as well as *post mortem* examinations were conducted following a strict protocol [36]. Three snakes were euthanized and examined pathologically on days 4, 16, 27 and 49. *Intra vitam* sampling included blood samples for further immunological studies [37] and tracheal washes/cloacal swabs for virus detection [36]. Three snakes from the placebo group were euthanized and examined pathologically on day 49.

Necropsy was conducted following published standards [59]. Gross pathology included the assessment of all inner organs. Samples of the lung tissue as well as the liver, the kidney, the small and large intestine, the pancreas, the spleen, the brain and the gonads were processed for histopathological examination following published standards. All lung samples were taken from the cranial aspect of the lung tissue. All tissue samples were evaluated histologically after fixation in 4.5% phosphate-buffered formalin, then embedded in paraffin, sectioned at 4μm, and stained with haematoxylin and eosin (H & E). Furthermore, for stereologic evaluation using light microscopy as well as transmission electron microscopy (TEM), lung tissue was taken from cranial, middle and caudal parts of the lung and immediately prepared for fixation, using 5% paraformaldehyde (for light microscopy) and 2.5% glutaraldehyde (TEM samples) each in 0.1 mol/l phosphate buffered saline (according to Starck [60], details listed there). For light microscopical morphological assessment, the samples were orientated as cross-sectional fragments through the organ, representing the tissue from the central lumen to the outer wall of the lung [61], and stained with methylene blue-thionine. TEM samples were post-fixed in 1% osmium tetroxide for 30 min, dehydrated using a graded series of ethanol and then embedded in Durcupan ACM resin (Sigma Aldrich, Hamburg, Germany). Sections were prepared in random orientation, and each of eight ultrathin slices were used for morphometry, using a Morgani digital transmission electron microscope (FEI Germany, Kassel, Germany). Capillary wall thickness was measured in five capillaries at each of five positions, using the orthogonal intercept method [15,62]. Based on these data, the harmonic mean thickness of the epithelium, the basal lamina and the endothelium, forming the air-blood barrier, was calculated (more details on the method and detailed results on the morphology and morphometric measurements given by Starck et al. [63]).

For bacteriological and mycological culture, the faveolar surface of the lung tissue was sampled with individually packed sterile microbiological swabs (Applimed, Freiburg, Switzerland) and immediately plated onto agar and *Salmonella* enrichment bouillon. As initial isolation media, Columbia blood agar (Oxoid, Wesel, Germany), MacConkey agar (Oxoid) and brilliant green agar (Oxoid) were inoculated with the samples and then incubated at 30°C for 24 hours under aerobic conditions. A mycological culture was performed on Sabouraud dextrose agar (Oxoid) as well as potato dextrose agar (Oxoid) and incubated at 30°C for up to 120 hours. Differentiation of bacteria was carried out using Gram staining, CrystalTube (BD Biosciences, Heidelberg, Germany), and MALDI-TOF-mass spectrometry (Bruker microflex LT mass spectrometer, Bruker BioTyper 1.1 software, Bruker Daltonik GmbH, Leipzig, Germany) [64]. For detection of *Salmonella* species, samples of lung tissue were inoculated into Rappaport Vassiliadis enrichment broth (Oxoid) and Selenit enrichment broth (Oxoid), incubated at 41°C for 48 h followed by culture on XLT4 agar (Oxoid) and brilliant green agar (Oxoid) at 41°C for 24 h. All *Salmonella* strains were serotyped at the German National Salmonella Reference Laboratory in Berlin. The classification of *Salmonella* subspecies and below the subspecies level was carried out according to the White-Kauffmann-Le Minor Scheme [65] by agglutination with O- and H-antigen specific sera.

Details on the virus detection methods and results are published elsewhere [36]. Intra vitam and post mortem samples were checked for the presence of ferlaviruses using both PCR and cell culture methods. Post mortem examinations demonstrated virus detection in 92% of all lung tissue samples.

### Assessment and scoring

In order to standardize the evaluation and to compare the results of different assessments and measurements between the groups, a scoring system was used for all pathological parameters evaluated in this study, and a total score was calculated for each individual snake, as well as an average score for the respective infection group. The scoring details are provided in Table 1.

**Table 1:** Score definitions for different pathological criteria (for pathological alterations, only those signs are listed as relevant that were found in this study). Further details on the classification of the scores are provided in the text.

Criterion	Score		
	0	1	2
Clinical status	No abnormalities detected	Mucus secretion choana, tracheal wash fluid contains visible particles	Secretion from trachea, respiratory sounds, respiratory distress, CNS abnormalities, apathy, spontaneous death
Lung macroscopy	Unremarkable respiratory tissue, no thickening, no mucus secretion	Colorless mucus secretion, reddening or mild thickening of lung tissue	Purulent or yellowish mucus secretion with visible particles, thickening and reddening of the lung tissue, mucus secretion in the air sac
Lung microscopy	No inflammatory reaction on histology slides, normal appearence of the faveoli, no mucus secretion	Inflammatory reaction consisting of mainly lymphocytes and plasmacells in the interstitium	Moderate or severe inflammatory reaction, incl. mainly heterophilic infiltrates in the interstitium, faveoli filled with fibrin, heterophils and histiocytes, inflammatory cells, granuloma formation
Other organs microscopy	No alterations possibly associated with infection	Indication of infection, including pigment nephrosis and/or hemosiderosis and/or hyperplasia of melanomacrophages in the liver	Inflammatory reaction in other organs: e.g. hepatitis, pancreatitis, splenitis
Lung morphology*	About 2/3 of air-blood barrier typical three-layered structure (epithelium, basal lamina, endothelium of approximately same thickness, type I pneumocytes with typical appearance as part of the air blood barrier, type II pneumocytes in spaces between capillaries,occasional inhomogeneous appearance of the basal lamina possible	Increasing thickness or hypertrophy of the type I pneumocytes and/or the endothelial cells and/or thickening of the basal membrane, only partial alterations with presence of lymphocytes	Transformation of pulmonary epithelium into a multilayered pseudostratified/stratified epithelium, type I cell transformation to mainly columnar cells with large nucleus and many mitochondria, lymphocytes at the base of the epithelium, activation and proliferation of type II pneumocytes, capillaries moved deeper into surrounding tissue, mucus and bacteria might be present in lumen, all samples affected
Air-blood barrier*	< 1.90 μm	1.90 to 5.10 μm	> 5.10 μm, or no measurement possible
Microbiological culture lung	No bacterial detection	Bacterial detection	-

*Based on Starck et al. [60]

Clinical signs were recorded according to a detailed protocol and scored according to severity for each animal during each examination. Each snake was assessed daily, with special emphasis on the general condition, behaviour, the respiratory system including the oral cavity (weekly, as well as on sampling days) and the central nervous system. Unremarkable snakes were active (when taken from their hiding places) and maintained normal position, the oral mucosa was bright without mucus, and less than six breathing movements were observed per minute. The tracheal opening was free from exsudate and closed except for breathing cycles. No respiratory sounds could be heard. Clear tracheal wash fluid was considered normal in those snakes still alive on days 4, 16, 28 and 49. Clinical status was scored from unremarkable (0) to severe clinical signs typical for ferlaviral disease (2).

In gross pathology, the lung tissue was assessed macroscopically for signs of inflammation. Scoring ranged from unremarkable (0) tissue, mild signs of pneumonia (1) to moderate/severe signs of pneumonia (2). Mild signs of pneumonia were defined by colorless mucus, reddening or mild thickening of lung tissue. Moderate to severe signs of pneumonia were defined as yellowish/flaky mucus, thickening and reddening of the lung tissue as well as mucus in the air sac. Microscopically, the lung tissue was scored based on the results of both the assessment of the routine histology slides as well as the slides prepared for light microscopy stereological assessment. The overall appearance of the faveoli was assessed, as well as inflammatory reactions and mucus secretions in the faveoli. Further, all other organs examined histologically were assessed for alterations as a possible consequence of a ferlavirus infection. Mild secretion and unspecific findings such as reddening of the lung tissue were conisidered mild alterations (1), whereas typical inflammatory reactions were scored as severe alterations (2).

The morphology of the healthy as well as the diseased ophidian lung cell structure has been described in detail [15]. Based on these descriptions, the ultrastructural morphology of the samples was assessed and scored from 0 (normal) to 2 (severe alterations).

The scores for the “air-blood barrier” were assessed based on the mean thickness of the air blood barrier measured in healthy and diseased Burmese pythons [15], and the difference in the thicknesses depending on the health status. Based on this information and the average value for the air-blood barrier found for three snakes from the placebo group in this study, a thickness up to 1.9 μm was considered normal (0), and a thickness of more than 5.1 μm (more than 425% of the normal value, or exceeding measurable range) as a severe alteration (2).

Microbiological results from lung tissue samples were scored with 0 or 1, with any bacterial or fungal detection scored as 1.

Statistical evaluation of the scoring results was conducted using the software SPSS 25.0 (IBM, Armonk, USA). Since an ordinal scoring system was used in this study, non parametric tests were chosen for comparison of the results obtained. For each of the different pathological criteria, the Pearson’s chi-squared test with exact p-value calculation was used to evaluate significant differences between the groups in general. In case of a significance, as post hoc tests the groups were then tested against each other using the same test, to check for statistical significances between the three individual virus strains used. To evaluate possible correlations between the scores for the different pathological ciriteria, Kendall’s tau b was calculated for all groups together, as well as Cohen’s kappa coefficient to measure the agreement between the scoring parameters. In case of a significance a post hoc calculation followed within each group. Statistic evaluation for significant changes between the sampling days (from day 4 to day 49) is only meaningful within each group but not for all groups together. However, as there are only about three samples for each sampling day, only a global test (Pearson’s chi-squared test with exact significance) was conducted, as statistic comparisons between the individual days were not expected to lead to meaningful results. For all calculations significance was assumed with p≤0.05, and a high significance level with p≤0.001. As the study intention was explorative, no adjustment for multiple comparisons was performed.

### Ethics statement

A detailed animal trial application listing all planned procedures was submitted to the national authority (Landesdirektion Sachsen) and discussed in the associated ethics committee (Tierschutzkommission Leipzig). The application was submitted with the identification number TVV 61/13 and was fully approved on Feb 26, 2014. The authorties approval is based on the following national laws and regulations: Tierschutzgesetz §8, Tierschutz-Versuchstierverordnung including the animal care and use protocols listed there.

## Results

### Phylogenetic analysis and pairwise comparison of the genome segments

Surface glycoprotein gene (F and HN) amplification and sequencing was successful for nine squamate ferlavirus isolates (S1 Table) of which two belonged to group A, six to group B and one to group C. Earlier reported partial L gene sequences were also extended, thus producing a contiguous genome segment with a consensus length of 5.3 kb for these isolates. The two lizard isolates Var-GER95 and Xeno-USA99 turned out to have identical sequences, therefore only the name of the latter is shown on figures and tables in this paper. The sequences were submitted to GenBank and have been assigned the accession numbers: MK241842 to MK241849.

**Fig 1:**
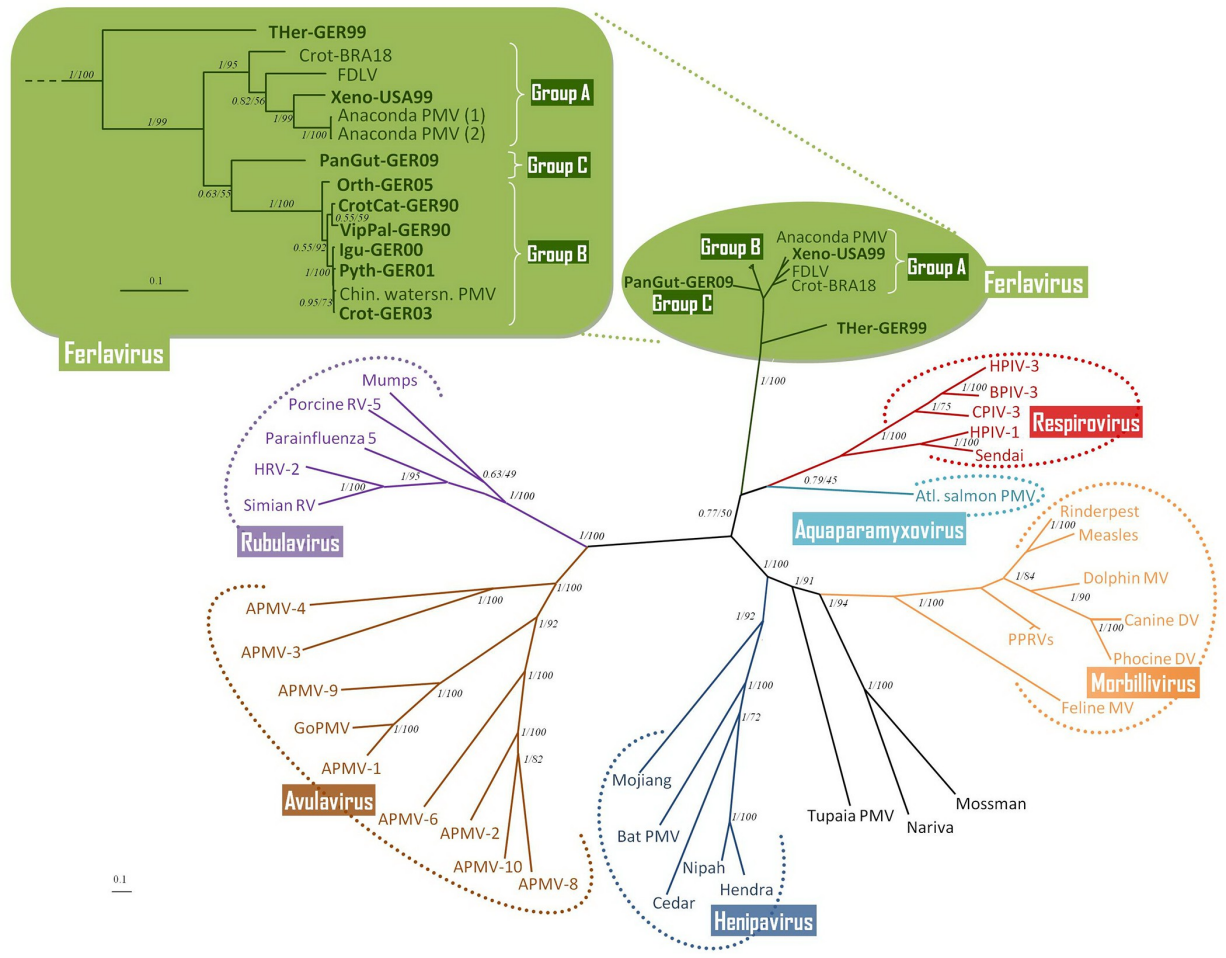
Combined phylogenetic tree of *Paramyxoviridae* based on the MAFFT alignment of the complete F, HN and partial L gene nucleotide sequences (5.3 kb). GBlocks editing resulted in a 2.7 kb alignment which was used for the phylogenetic calculations. Genus *Ferlavirus* is enlarged in the top left section, own sequences are highlighted in bold. Two applied algorithms (Bayesian/Raxml) converged in the resulting tree topology. The former was taken for this combined representation. Bayesian posterior probability and ML bootstrap percentage values at the branch nodes indicate the robustness of these calculations. Abbreviations and accession numbers (in bracketes) listed according to genera: *Aquaparamyxovirus*: Atlantic salmon paramyxovirus (EU156171); *Avulavirus*: APMV = Avian paramyxovirus, APMV-1 (KY284861), APMV-3 (EU782025), APMV-4 (EU877976), APMV-6 (AY029299), APMV-8 (FJ215864), APMV-9 (EU910942), APMV-10 (NC_025349), GoPMV = Goose PMV (AF473851); *Ferlavirus*: Anaconda PMV (KJ956407 & KJ956408), Chinese watersnake PMV (MG600060), Crot-BRA18 = Brazilian Crotalus PMV isolate (MH411104), FDLV = Fer-de-lance virus (AY141760); *Henipavirus*: Bat PMV (NC_025256), Cedar virus (NC_025351), Mojiang virus (KF278639), Hendra virus (AF017149), Nipah virus (KY425655); *Morbillivirus*: Canine distemper virus (KU578257), Dolphin morbillivirus (AJ608288), Feline morbillivirus (KR014147), Measles virus (AB016162), Phocine distemper virus (NC_028249), PPRV = Pest de petit ruminants virus (KM816619), Rinderpest virus (JN234008); *Respirovirus*: Sendai virus (AB005796), HPIV-1_AF457102_(part), BPIV-3 = Bovine parainfluenza 3 virus (Y00114), HPIV-3 = Human parainfluenza 3 virus (MF554733), CPIV-3: Caprine parainfluenza 3 virus (KT215610); *Rubulavirus*: HRV-2 = Human rubulavirus 2 (NC_003443), Human parainfluenza 5 virus (NC_006430), Mumps virus (AB040874), Porcine rubulavirus 5 (NC_009640), Simian rubulavirus (X64275), *Unassigned to genus*: Mossman virus (AY286409), Nariva virus (FJ362497), Tupaia PMV (AF079780).

This genome segment was used to resolve the phylogenetic kinship of squamate ferlaviruses with other paramyxoviruses available in GenBank (Fig 1). No sign of recombination was found among Ferlaviruses using this alignment. For the use of phylogenetic calculations the alignments were edited to delete possibly distorting gaps using a GBlocks Server or manually, optimizing for a possibly better resolution among ferlaviruses. These edited alignment (GBlocks: 2696 nt, manual: 4763 nt) were subjected to phylogenetic calculations, testing several methods. The resulting trees, based on maximum likelihood, distance-matrix based and Bayesian inference based analyses, were greatly comparable to each other and to those of previously published analyses [11,24,25] (Fig 1). All applied programs consistently supported the separation of two squamate groups with high confidence values: a rather heterogenous group A and more homogenous group B. The exact position of the PanGut-GER09 (group C) sequence varied depending on the methods used, due to the lack of sequence data from another group C representative (e.g. HoBuc-HUN09), however, when it clustered closer to either group A or group B, its position was only supported by low confidence values. Athough it is not the subject of this present paper, we also included the unpublished homolog sequence of the tortoise PMV isolate (THer-GER99) into the phylogenetic comparison which, as suggested previously [11], clustered as a sister group to the squamate ferlaviruses in all of the trees, supported with high confidence values.

**Fig 2:**
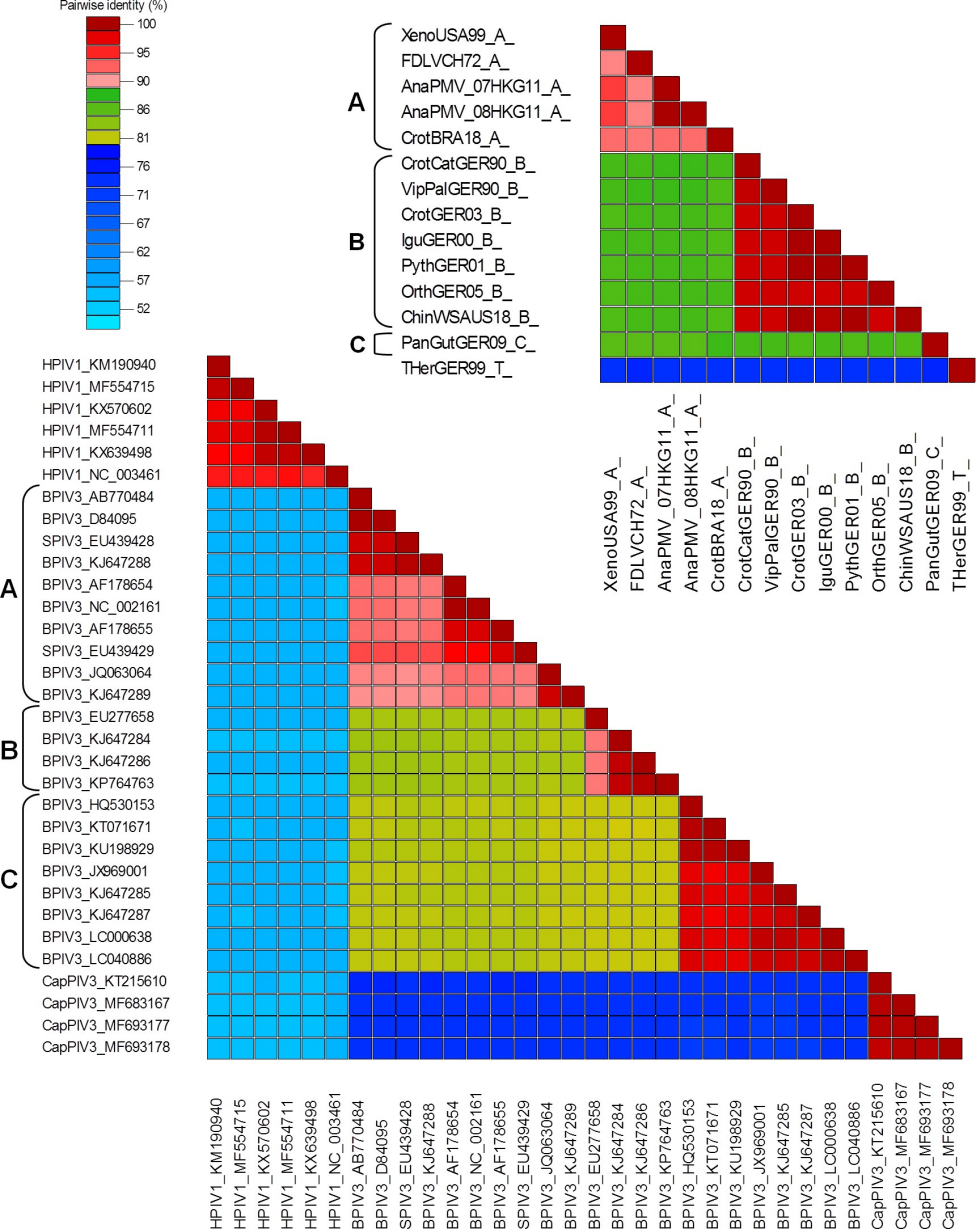
**Colored distance matrix of MAFFT pairwise nt alignment based comparison (SDT analysis) of the F-HN-L(part) sequences (5.2 kb) of the available ferlaviruses (top right) and selected respiroviruses (bottom left).** Cut-off values were set to 80–90% (see scale on top left), which resulted in the clear separation of the (geno)group within virus species in both genera. (Geno)groups are indicated with A, B and C to the left of the virus identifiers. These identifiers are derived from the virus name abbreviation and GenBank accession number in case of the respiroviruses. Identifiers of ferlaviruses are derived from the short isolate names used on the other figures, containing the host name, country (capitalized) and year of isolaton (last two digits), followed by group code. E.g. FDLVCH72_A codes Fer-de-lance virus from Switzerland {CH} in 19{72} belonging to group A.

Assigning possible group demarcation criteria for the *Ferlavirus* genus and comparing it with those of another, more thoroughly studied PMV genus, delineated cut-off values of 80 and 90% for the separation of the viral species and the (geno)groups, respectively. Pairwise alignment based comparison, using the SDT programme of the 5242 nt genome segments of 14 ferlaviruses with each other and the same analysis of the homologous parts of 32 selected respiroviruses (among them members of three BPIV-3 genogroups) revealed that these values can be applied to both genera (Fig 2). Performing the same analysis on the complete genomes of the selected respiroviruses gave the same results as that for their ca. 5.2 kb segments.

### F protein motif diversity and protein models

The three nucleotide long intergenic regions between the F, HN, and L genes of the newly sequenced ferlavirus isolates were typically CCU for all group A and B isolates and CUU for the PanGut-GER09 isolate of group C. Gene start and stop motifs were highly conserved within all ferlavirus isolates. Primary analysis of protein sequences revealed high conservation of motifs, although at some points, individual residues that differed from those of the other squamate isolates could be found (S1 and S2 Figs).

Concerning the fusion protein, at the N-terminus, the signal peptide (SP) was predicted at residues 1–19, 1–18, and 1–18 aa for our group A, B and C isolates, respectively. Interestingly, both Phobious and SignalP 4.1 sites failed to identify the presumed SP in the sole group C member (PanGut-GER09). However, based on SignalP 3.0 analysis, we presume its position. In general, this motif was not conserved and had several hydrophilic residues substituted with other hydrophilic ones.

The next predicted major motif is the furin recognition site, at position 107–110. This *in sensu stricto* domain contains four amino acids upstream (from -4 to -1) from the cleavage site (CL) which is conserved for all members of **groups A** and **B** (R-E-K-R), with a slight variation in the **group C** member (R-G-K-R). There are conserved and non-conserved substitutions in the *sensu lato* regulatory region of the recognition site, at positions 101–106 (-10 to -5 relative to the CL), which mark the separate groups. Concerning our isolates, this sequence was, for the **group A** members: S-K-V-S-T-K, for **group B**: P-K-T-L-S-R/K, while for the sole **group C** member: L-K-T-P-A-K. The subsequent motif, right downstream of the cleavage site, at position 111–135 of the F0 protein, is the fusion peptide (FP), which was found to be conserved in all of our isolates (S1 and S2 Figs).

We also identified the two major heptad repeats (HRA and HRB) and two further presumptive HR regions (HR3 and HR4) within the F0 protein in our ferlavirus isolates, as well as in the sequences available in GenBank. The HRA and -B are six-time repeats (at positions 145–186 and 461–502), while HR3 and HR4 are three-time repeats (pos. 263–283 and 80–100) of seven amino acid long patterns (positions ‘a–g’, S1 Fig). A leucine zipper motif (LZ) was predicted along HRB, containing hydrophobic amino acids leucine (L) or isoleucine (I) at position ‘a’. All four HR domains detected were highly similar among our isolates, with only 4.7–14.2% varying residues (4/21 aa, 2/42 aa, 2/21 and 6/42 aa; for HR4, HRA, HR3 and HRB, respectively). These changes were conserved or semi-conserved, appearing both on the neutral and charged faces of HR4 and HRA and only on the charged face of HRB helices. The coiled coil prediction did not forcast a definitive single helical structure in the region of HR3, whereas it was shown for the other three HR regions. Some changes showed a rather group-specific distribution, along with the homologs from GenBank included in the comparison (S1 Fig). A single residue within HRA (pos. 157), another in HRB (pos. 480) and two in the surrounding regions (pos. 135, 251) with dissimilar aa changes delineated the (geno)groups. The last identifiable motif of the F protein was the transmembrane anchor, which was predicted at position 502–520, near the C-terminus. This motif contained changes at 10 residues (52.6%), most of them remaining hydrophobic, with no specific pattern for the 3 ferlavirus groups. Using NetNGlyc 1.0, two possible glycosylation sites (N-G) were detected at positions 435/436 and 442/443.

**Fig 3:**
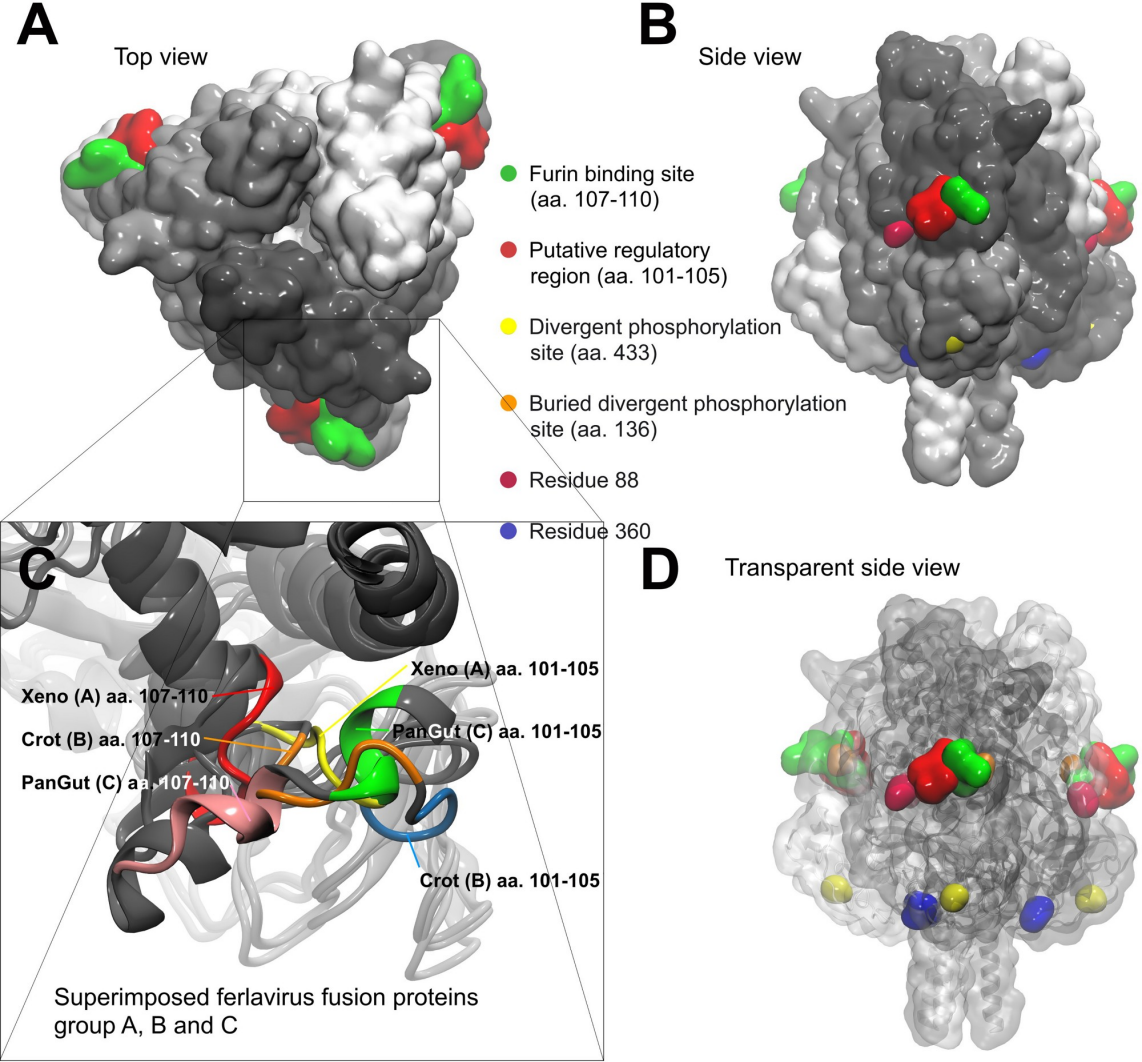
Ferlavirus fusion protein trimer models. The furin binding site (green) and the newly described putative regulatory region (red) located at the edges of the triangle shaped fusion protein trimer complex. The divergent predicted phosphorylation sites are also indicated on the models. These sites can be seen from top (A) and side views (B) and a transparent side view (D) was rendered to see the buried divergent phosphorylation site. Variations of two residues (88 and 360) significantly alter the electrostatic charge distribution around the furin binding site. Panel C shows the structural differences between the three studied ferlavirus groups around the furin binding site.

**Fig 4:**
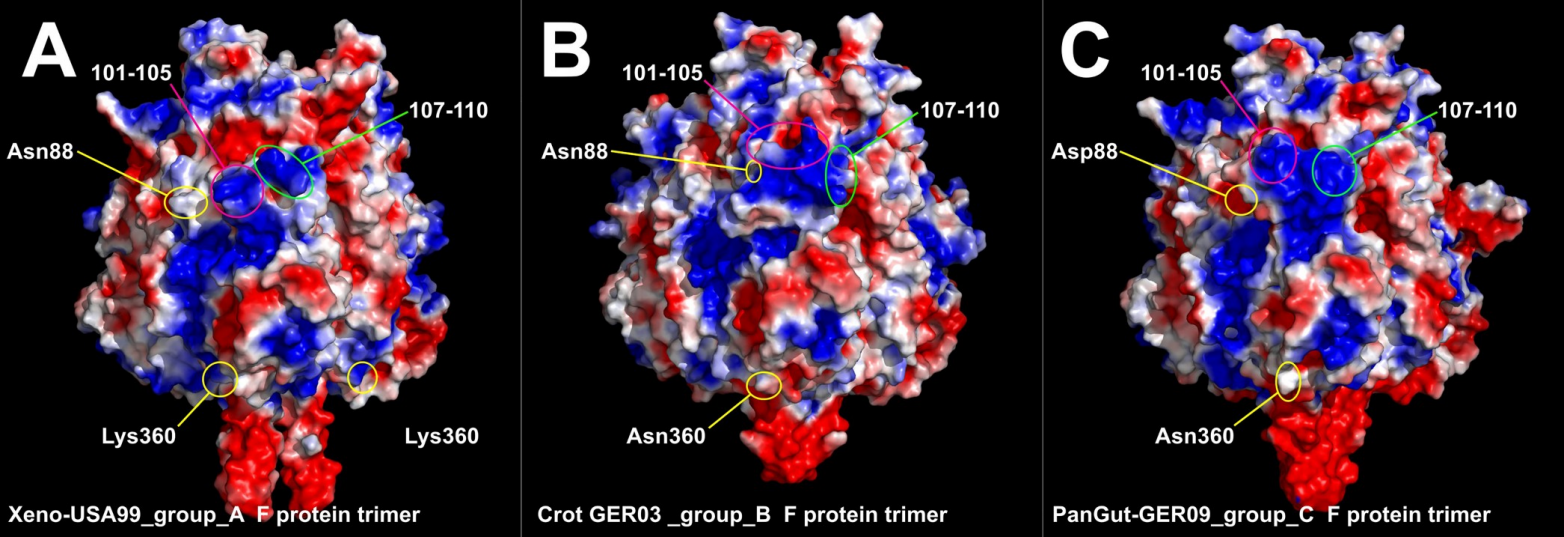
Electrostatic surface view of ferlavirus fusion protein dimers. Red represents regions with potential values less than -2.0 kT; white represents 0.0; blue shows regions greater than +2.0 kT. Amino acids in position 88 and 360 are indicated in panel A, B, and C, respectively.

3D protein models were also created so as to visualize the predicted structural differences between the well separated ferlavirus A, B and C group members. Additional comprehensive predictions were run to identify those putative phosphorylation sites which might parallel the phylogenetic classification into the three groups. Only one significant conformational change could be localized shortly before the furin recognition site (Fig 3), which was suspected to serve as a ‘regulating region’ or extension of the furin recognition site. Moreover, this structurally divergent small region (aa. 101–105) was detected as a multiple phosphorylation site which shows different patterns in the A, B and C groups. We found three additional putative phosphorylation sites, in parallel with the grouping, located in the cleaved part (F1) of the F protein (Fig 3D). But the first of them (aa 136) only becomes accessible for the kinase after furin cleavage. Interestingly, we detected two positions (88 and 360) in the ferlavirus amino acid sequence alignment which significantly influence the electrostatic charge distribution around the furin binding site (Fig 4). For instance, Asp88 in the PanGut-GER09 isolate wedges a large negative charge shortly before the furin binding site (Fig 4C). While Lys360 in the Xeno-USA99 isolate introduces a positively charged surface area at the bottom of the fusion protein trimer (Fig 4A).

### HN gene motif diversity and protein models

In the HN protein, the first 27 aa were identified as the cytoplasmic tail, which is not greatly conserved, with 15 of these residues changed among the different viruses from groups A, B, and C both from our study and compared to other ferlavirus sequences in GenBank (S2 Fig). The following residues (28–50) represent the transmembrane domain with mostly hydrophobic residues with a few conserved variations (V/I/L) among the isolates, and one non-conserved change (A→G) compared to the FDLV sequence. The next motif, at positions 50–115, which constitutes the stem in the HN protein, showed a number of non-conserved substitutions as well, distributed in a group-specific pattern (e.g. positions: 59, 63, 66, 74, 78, 88). Following the stem region is the head motif, which was found to be more conserved among the squamate viruses. The next are the amino acids (246–251) representing the sialic acid binding site (N-R-K-S-C-S).

The C-terminal region starting from position 300 was very conserved, except for a few individual positions where non-conserved substitutions were detected particularly in the PanGut-GER09 isolate (S2 Fig). The sequence G-A-E-G-R at position 401–405 was very conserved with a maximum variation in 1 position (80–100% identity) in the members of the genera ferla-, respiro-, avula-, and rubulaviruses, and all cysteine residues were completely conserved for all ferla- and respiroviruses (S3 Fig). Two probable glycosylation sites were detected for all ferlavirus isolates at positions 128 and 163.

**Fig 5:**
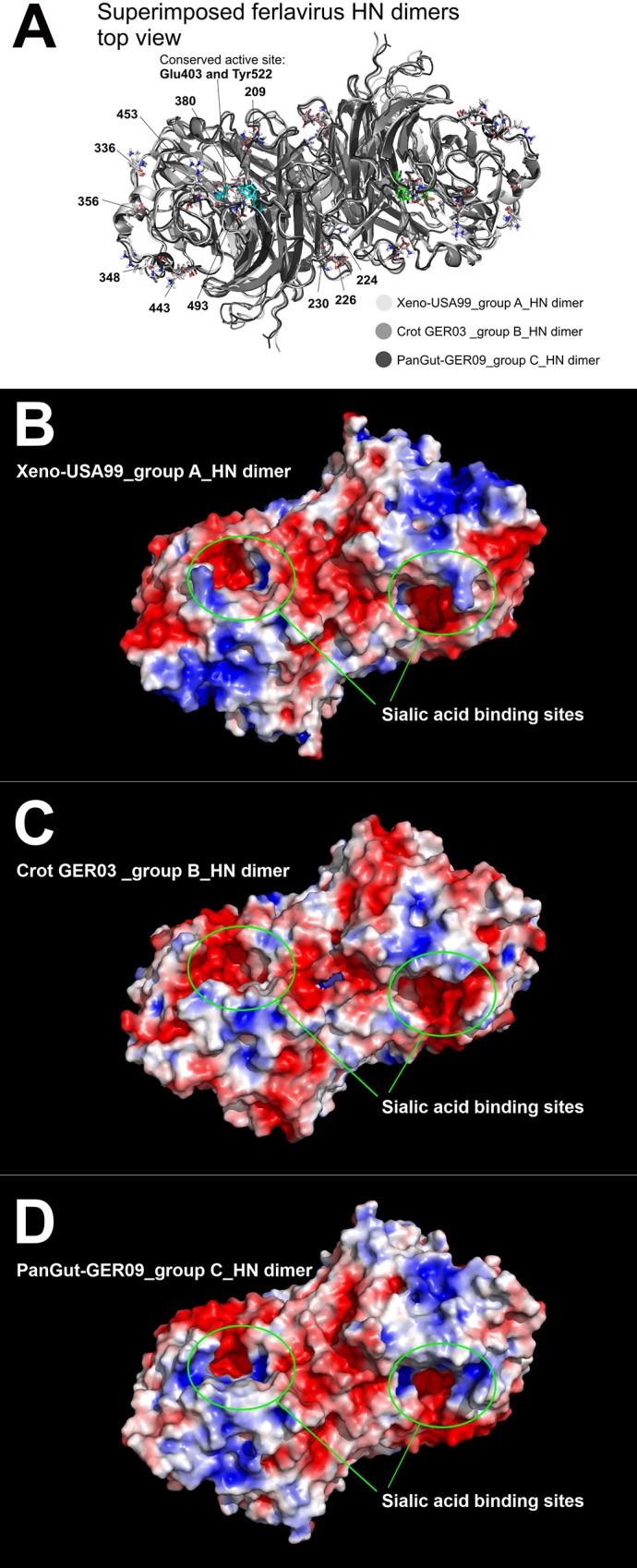
Ferlavirus hemagglutinin-neuraminidase dimer models. **Superposition of the three ferlavirus HN proteins reveals tight structural similarity (A).** Those residues that influence the electrostatic charge patterns are indicated on the figure. Electrostatic surface view of ferlavirus HN dimers (B, C, and D). Red represents regions with potential values less than -2.0 kT; white represents 0.0; blue shows regions greater than +2.0 kT.

In our standard 3D modeling study, we did not find significant structural differences between the three ferlavirus group members. More comprehensive analysis shed light on significant electrostatic charge distribution differences on the distinct ferlavirus HN protein surfaces (Fig 5). Basically, eleven amino acid positions (Fig 5A) were identified which influence the HN protein charge patterns. The catalytic residues are completely conserved at the active site (Glu403 and Tyr522) but different electrostatic patterns can be identified at the entrance of the binding pocket (Fig 5B, 5C and 5D). In addition, possible phosphorylation would alter the roles of the HN proteins by changing their electrostatic patterns. Eleven divergent phosphorylation sites were predicted on the modeled region of the HN proteins at the following aa positions: 97, 143, 194, 228, 283, 295, 301, 336, 350, 353 and 453 (S2 Fig).

### Pathogenesis following experimental infection

Clear differences were found in the development of clinical signs as well as pathological alterations in the lung tissue depending on the virus strain used for infection. The results are described for each trial group individually, followed by a comparison of the groups and statistical evaluation of the results. For each individual snake, the day of the post mortem examination and the scores according the scheme described in Table 1 are presented in Table 2.

**Table 2:** Evaluation scores given for each animal and each criterion. Except for the criterion “lung microbiology” (0 –unremarkable, 1 –remarkable), the scores were given as follows: 0 –unremarkable, 1 –moderate change, 2 –severe changes.

Necropsy day	ID	Clinical signs	Lung macroscopy	Other organs	Lung histology	Lung electron microscopy	Air blood barrier	Lung microbiology	TOTAL SCORE	AVERAGE PER DAY	AVERAGE TOTAL
**GROUP A „Xeno-USA-99“**											
4	A1	0	0	1	1	0	0	0	**2**	1.7	4.3
	A2	0	0	1	0	0	0	0	**1**		
	A3	0	0	1	1	0	0	0	**2**		
16	A4	0	0	1	1	0	0	1	**3**	3.0	
	A5	0	0	1	1	0	0	1	**3**		
	A6	0	0	1	1	1	0	0	**3**		
28	A7	0	1	1	1	2	2	0	**7**	5.7	
	A8	0	1	1	2	2	2	0	**8**		
	A9	0	0	1	1	0	0	0	**2**		
49	A10	1	0	1	2	2	1	0	**7**	6.7	
	A11	1	2	2	2	2	2	0	**11**		
	A12	0	0	0	1	1	0	0	**2**		
AVERAGE		0.2	0.3	1.0	1.2	0.8	0.6	0.2			
**GROUP B „Crot-GER09“**											
4	B1	0	0	2	1	0	0	0	**3**	2.0	8.7
	B2	0	0	0	0	0	0	0	**0**		
	B3	0	0	2	1	0	0	0	**1**		
16	B4	0	2	2	2	2	2	1	**11**	9.7	
	B5	0	2	0	2	2	2	0	**8**		
	B6	0	2	2	2	2	2	0	**10**		
27	B7	2	2	0	2	2	2	1	**11**	10.3	
28	B8	2	2	0	2	2	2	1	**11**		
	B9	2	2	0	2	1	1	1	**9**		
33	B10	2	2	2	2	2	2	1	**13**	12.7	
35	B11	2	2	2	2	2	2	1	**13**		
	B12	2	2	2	2	2	1	1	**12**		
AVERAGE		1.0	1.5	1.0	1.7	1.4	1.3	0.6			
**GROUP C „PanGut-GER09“**											
4	C1	0	0	0	1	0	0	1	**2**	2.5	5.4
	C2	0	0	1	1	0	0	0	**2**		
	C3	0	0	1	1	0	0	0	**2**		
	C4	0	1	2	1	0	0	0	**4**		
16	C5	0	0	2	1	2	1	0	**6**	5.0	
	C6	0	0	2	1	1	0	0	**4**		
27	C7	2	2	0	2	2	2	1	**11**	8.0	
28	C8	0	0	0	1	1	0	0	**2**		
	C9	1	2	2	2	2	2	0	**11**		
36	C10	2	2	0	2	1	0	1	**8**	7.0	
37	C11	2	2	0	2	2	2	1	**11**		
49	C12	1	0	0	0	1	0	0	**2**		
AVERAGE		0.7	0.8	0.8	1.3	1.0	0.6	0.3			
**PLACEBO**											
49	P1	0	0	0	0	0	0	0	**0**	0.3	0.3
	P2	0	0	0	0	0	0	0	**0**		
	P3	0	0	0	1	0	0	0	**1**		

#### Placebo group

**Fig 6:**
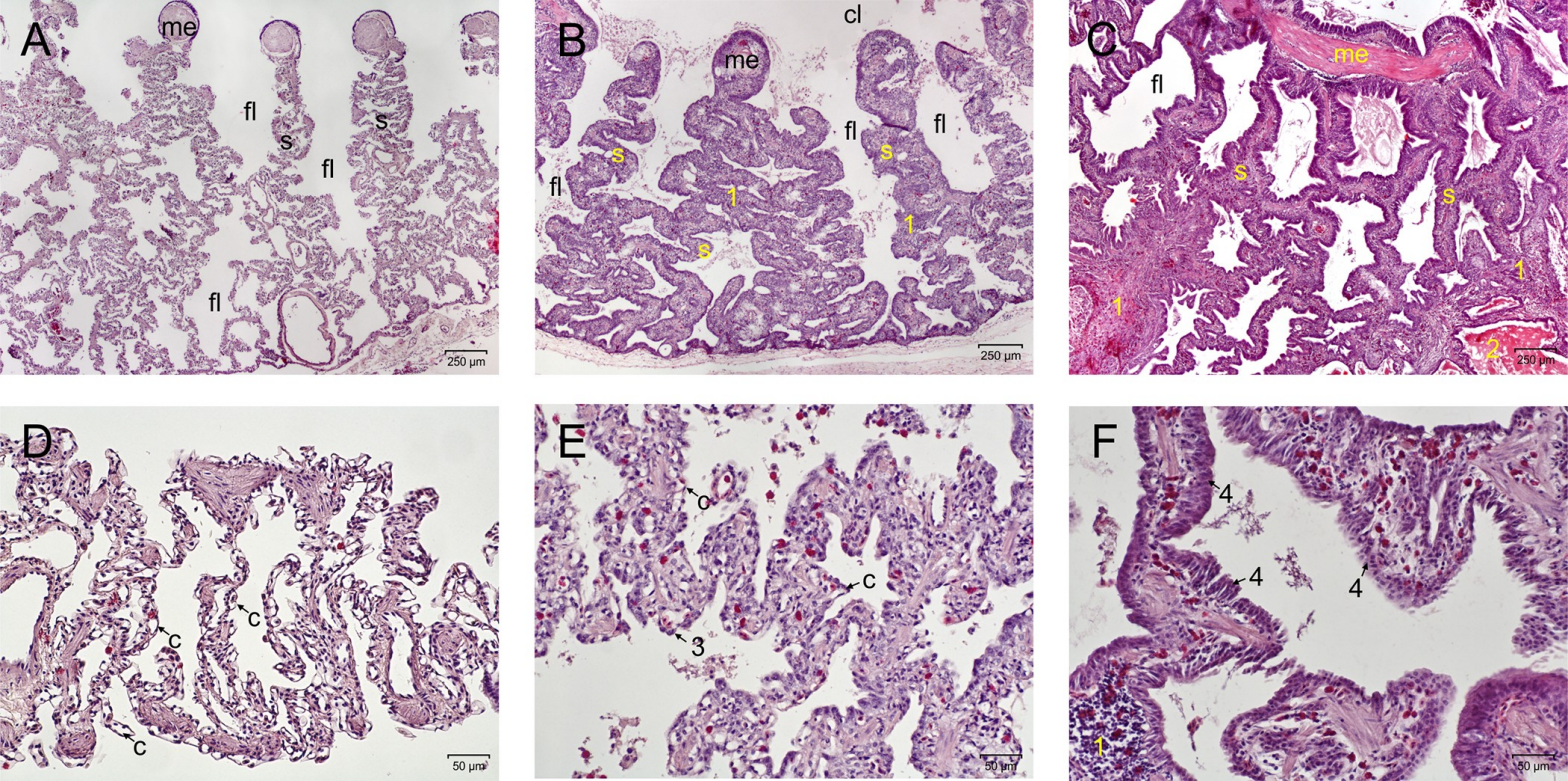
**Sections of the lung, light microscopy, H&E stain, A-C: 40x magnification; D-F: 200x magnification, day post infectionem.** (A,D) Score 0, snake No. P1, day 49. Demonstration of a cross section through an unremarkable lung. The myoelastic inner border of the lung tissue and faveoli lead to the respiratory tissue along the septae. Capillaries are bulging into the air spaces, demonstrating a thin air-blood-barrier. (B,E) Score 1, snake No. A4, day 16. Demonstration of a cross section through a ferlavirus infected lung, moderate changes. The septae appear thickened and initial transformation of the lung epithelium can be seen. Capillaries are still close to the surface, but the epithelial cells are starting to transform (3). Infiltration of lymphocytes and plasma cells in the septae (1). (C,F) Score 2, snake No. B11, day 35. Demonstration of a cross section (not complete) through a severely affected lung. The septae are massively thickened, the faveoli narrowed and partially filled with debris (2). Massive thickening and infiltration of heterophils, lymphocytes and plasma cells in the interstitium (1), transformation of the respiratory epithelium into a multilayered epithelium (4). (c–capillary; cl–central lumen; fl–faveolus; me—myoelastic tissue; s—septum).

The placebo group (all six snakes) remained clinically unremarkable over the entire trial period. The three animals that were selected randomly for necropsy and pathological examination (only the scores for these are given in Table 2) were all scored as unremarkable (total score 0, see Table 2, Fig 6A and 6D), except for the histological examination in one snake, which revealed a mild lymphoplasmacytic infiltration (score 1). In this group, no virus was detected in any organ or sample [36].

#### Group A

In this group, only two snakes showed moderate clinical signs (score 1), consisting of a tracheal wash sample containing visible particles on day 28 (A10), and a low amount of mucous secretion in the oral cavity on day 49 (A11). Over the 49 day trial period, no snake died spontaneously or had to be euthanized. Macroscopic evaluation of the lung showed a moderate inflammatory reaction (score 1) in two animals, resulting in a mild thickening and reddening of the tissue as well as a foamy mucus secretion, and a severe inflammatory reaction in one snake. Histologically, only one snake was unremarkable, but in eight animals, a mild multifocal lymphoplasmocytic infiltration of the interstitium could be seen (score 1, Fig 6B and 6E). Three snakes demonstrated moderate to severe heterophilic and lymphoplasmocytic infiltrates in the interstitium and heterophils as well as fibrin in the faveolar spaces (score 2). In ten snakes, hemosiderosis and pigment nephrosis was found in other organs (score 1), whereas one snake additionally suffered from a pancreatitis (score 2, A11).

**Fig 7:**
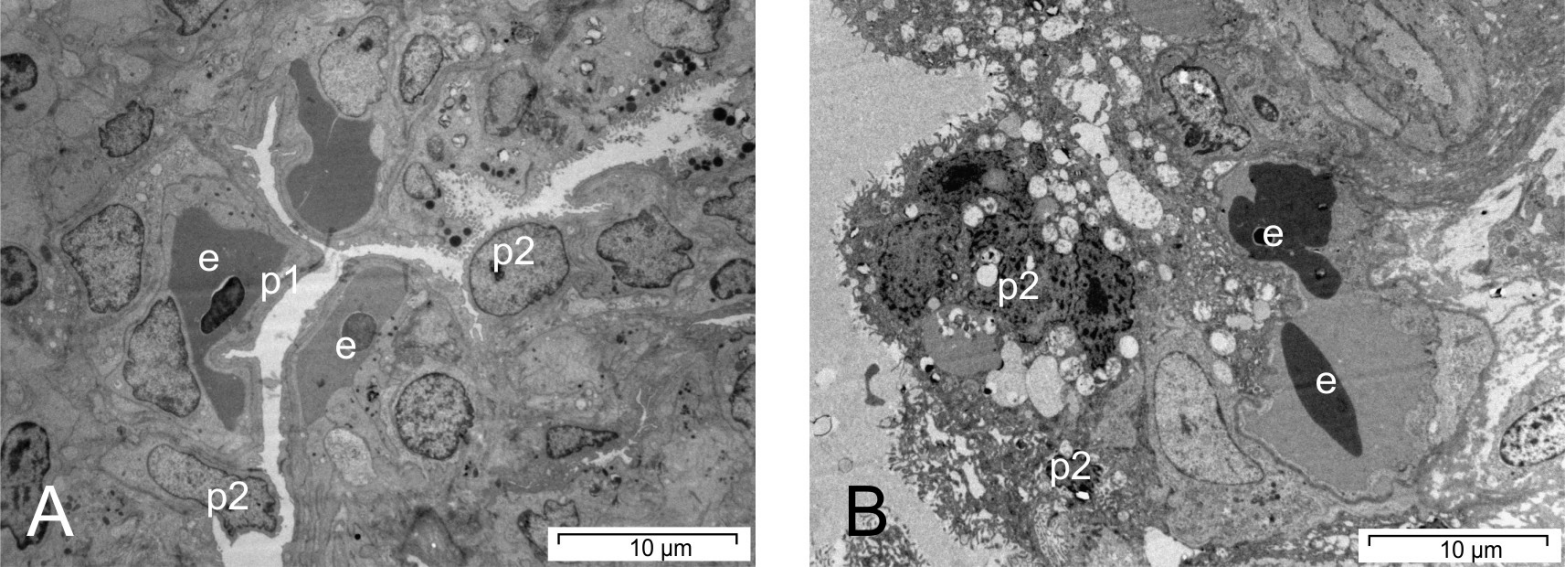
Sections of a lung, transmission electron microscopy of the respiratory epithelium. (A) Score 1, snake No. A12, day 49. Beginning transformation of the lung type-I epithelial cells into type II- pneumocytes. (B) Score 2, snake No. B4, day 16. Complete transformation of the epithelium into a multilayered epithelium consisting of activated type-II pneumocytes. (e–erythrocyte; p1—type-I pneumocyte; p2—type-II pneumocyte).

**Fig 8:**
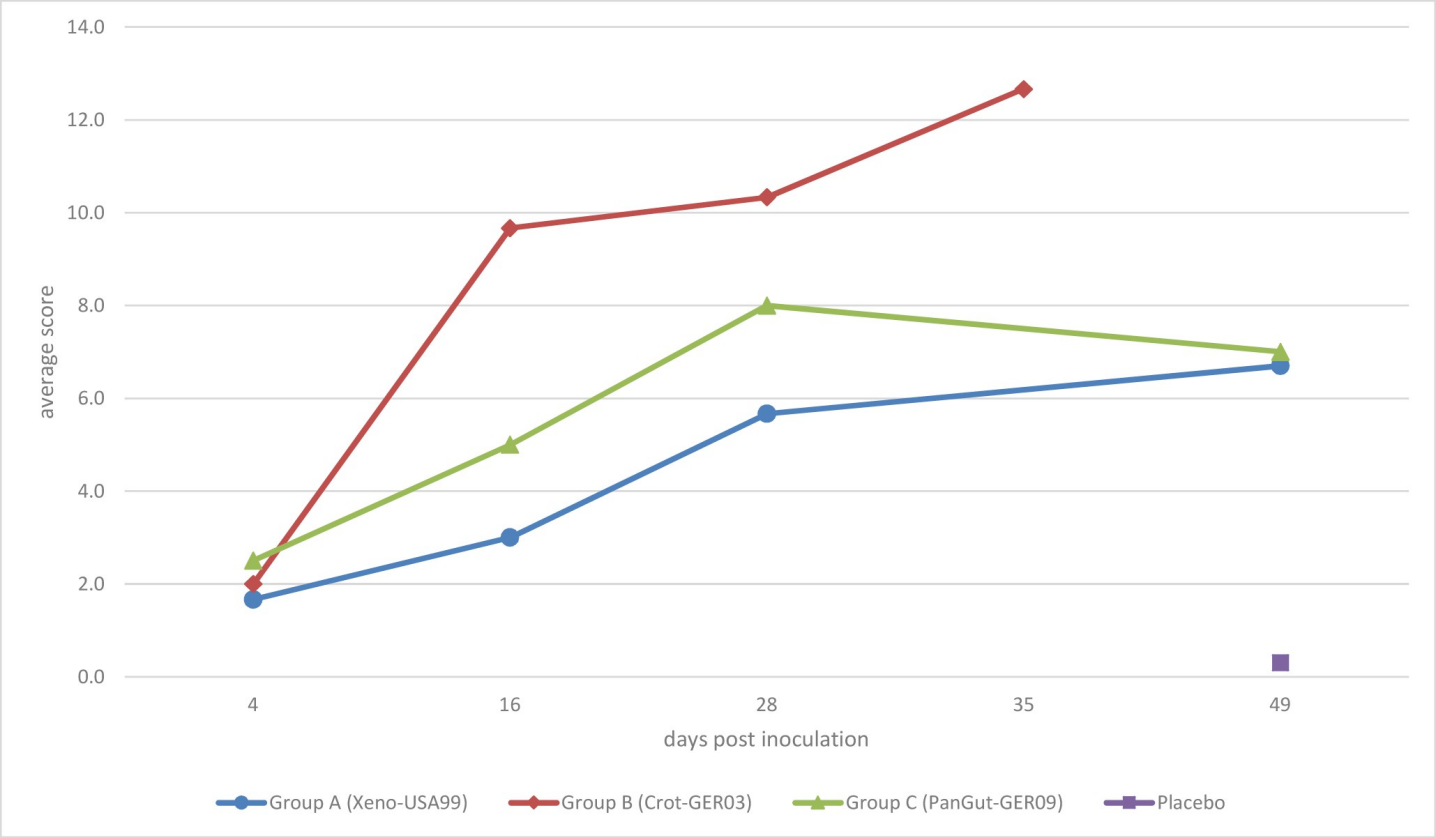
**Evaluation scores after inoculation with a genogroup A, B or C ferlavirus strain.** Each point is the average of the scores for all criteria for three animals that died by or were euthanized on the given day post inoculation (group C: day 4–4 snakes, day 16–2 snakes). Snakes in group B showed an earlier onset of pathological findiungs and a more dramatic course of disease, resulting in an early end of the study on day 35 post infection.

The morphology of the lung cells was unremarkable in six snakes, whereas in two snakes, type II pneumocytes changed their appearance and started to overgrow the capillaries, and the endothelial as well as basal lamina appeared slightly thickened (score 1, Fig 7A). In four snakes on days 28 and 49, the basal lamina was severely thickened, and the capillaries were mostly overgrown by type II pneumocytes (score 2). In those four snakes, the air-blood barrier was moderately (2.40 μm, score 1) or severely thickened (no measurement possible, score 2). The average score increased from sampling day 4 (1.7) continuously to sampling day 49 (6.7), with an average score of 4.3 (see Table 2 and Fig 8).

Microbiologically, no bacteria were cultured from the lung tissues of ten snakes. *Salmonella* were cultured from two snakes, (A4: *Salmonella* Kottbus 6,8:e,h:1,5, A5: *Salmonella* ssp. IIIa 40:z4,z23:-).

#### Group B

**Fig 9:**
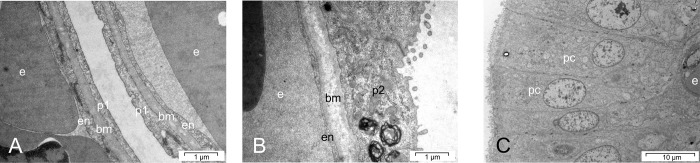
Sections of a lung, transmission electron microscopy of the air-blood barrier. (A) Score 0, snake No. C4, day 4. Demonstration of normal air-blood barrier. (B) Score 1, snake No. C5, day 16. Demonstration of an increasing thickness of the basal lamina and the epithelium. (C) Score 2, snake No. B8, day 28. Transformation of the epithelium into high prismatic cells and massive increase of the air-blood-barrier. (bm = basal lamina, en–endothelium, e–erythrocyte; p1—type-I pneumocyte; p2—type-II pneumocyte, pc = high prismatic transformed epithelial cell).

Six snakes (B7-B12) demonstrated severe clinical signs (score 2), starting from day 16 (B7, respiratory sounds, snake died on day 27), ongoing until the end of the trial. Signs included purulent secretion (B9), tracheal wash samples containing visible particles (B9-B12), respiratory sounds (B12), apathy (B11), abnormal position (B12), and sudden death (B7, B8, B10). Two snakes had to be euthanized due to their clinical condition (B11, B12). Lung macroscopy demonstrated a severe inflammatory reaction in all snakes from day 16 until the end of the study, resulting in severe thickening and reddish discoloration of the tissue, and yellowish stringy secretion in the lung lumen and partially also the air sacs. Histologically, snakes were only unremarkable on day 4. In all other snakes, a severe inflammatory reaction, consisting of a severe heterophilic and lymphoplasmocytic infiltration of the interstitium as well as fibrin in the faveolar spaces was found (score 2, Fig 6C and 6F). Hemosiderosis and pigment nephrosis was found in five snakes (B1, B3, B10-B12), five snakes demonstrated an acute non-purulent hepatitis (B1, B4, B6, B11, B12). A reactive splenitis was found in two snakes (B10, B11), whereas one snake each demonstrated acute catharralic enteritis (B3), epicarditis (B4) and an acute necrotizing pancreatitis (B12). Morphologically, snakes on day 4 were scored 0. All other snakes except for one were assessed to have severe morphological changes (score 2). These included capillaries that were fully overgrown by layers of type-II-pneumocytes (Fig 7B), making measurements often impossible. The endothelium also appeared thickened in some cases. Large inclusion bodies were seen in one snake (B8). The air-blood barrier was assessed to be normal in three snakes, moderately increased in two snakes, while measurements were not possible in seven snakes, due to the size (score 2, Fig 9C). The average score increased from sampling day 4 (2.0) continuously until the end of the study (12.7), with an average score of 8.7 (see Table 2 and Fig 8).

Microbiologically, bacteria were isolated from the lung tissue in seven snakes. This included mainly *Salmonella* serovars (B4: *Salmonella* ssp. IIIa 41:z4,z23:-; B7, B8, B12: *Salmonella* ssp. IIIb 48:l,v:1,5; B10: *Salmonella* ssp. IIIb 14:z10:z; B12: *Salmonella* Georgia 6,7:b:e,n,z15). *Citrobacter freundii* (B9) and *Klebsiella pneumoniae* (B10, B12) were also isolated.

#### Group C

Two snakes demonstrated mild clinical signs (score 1), consisting of mild mucous secretion in the oral cavity, in three individuals (C7: tracheal wash sample containing particles, C10: tracheal wash sample containing particles, followed by mucous secretion in the oral cavity and worsening of the general condition, C11: tracheal wash sample containing particles), clinical signs were assessed as severe. Three snakes died (C4: day 4 following anesthesia for blood sampling, C7: day 27, C11: day 37) and one had to be euthanized (C10: day 36).

Macroscopic evaluation of the lung demonstrated a mild reaction in one snake, and severe alterations in four snakes from day 27, consisting of severe tissue thickening and presence of large amounts of whitish to yellowish particulate to foamy or stringy secretions. In two cases, (C9, C11), the air sacs were filled with particulate to cheesy material. Histologically, only one snake (day 49), was unremarkable, whereas seven individuals demonstrated mild and four severe lung tissue alterations. A pigment nephrosis was found in four snakes (C2-4, C6), three snakes demonstrated a splenitis (C4-C6), and one animal suffered from an acute non-purulent hepatitis (C9). Morphological assessment of the lung tissue was unremarkable in four snakes, whereas in four snakes each the alterations were assessed to be mild or severe. The air-blood barrier was normal in eight snakes (Fig 9A), mildly thickened in one snake (Fig 9B) and severely increased in three animals. The average score increased from sampling day 4 (2.5) to day 28 (8.0), followed by a slight decrease that was caused by one almost unremarkable snake on day 49. The average score was 5.4 (see Table 2 and Fig 8).

No microorganisms were isolated from the lung tissue in eight snakes. *Citrobacter freundii* was isolated from four snakes (C1, C7, C10, C11). *Salmonella* Infantis 6,7:r:1,5 was also isolated from individual C1.

### Comparison and statistical evaluation

The scores of all individuals were compared between the groups for each evaluation category as well as for the overall score. Significant differences were found for the lung macroscopy, the other organ scores as well as the overall score. For these parameters, the lung macroscopy findings were significantly different between groups A and B (p = 0.003), as were the overall scores between group B and C (p = 0.037). For the other organ scores, group A was significantly different from groups B (p< = 0.001) and C (p = 0.004).

For all animals in the study (and in case of a significant correlation also within each infection group), correlations between the scores for the different evaluation criteria were calculated. Furthermore, the degree of agreement (exactly matching scores) was calculated using Cohen’s kappa test. In general, for most significant correlations, the level of agreement was also found to be significant, normally to a lower degree. For all snakes together, all scores correlated significantly except for the correlation between findings in other organs and any other scores. Further, two correlations were found for the lung microbiology scores (to lung macroscopy (r = 0.512) and lung histology (r = 0.483)), as well as a very weak correlation to clinical signs (r = 0.063). The highest correlations were found between the lung macroscopy, histology, electron microscopy and lung tissue barrier findings, each correlation ranging from r = 0.674 to r = 0.841. Significant correlations were also found between these scores within the groups. These significant correlations were strongest (r = 0.783 to r = 0.965) and most often found (9) in group B, and in comparison weakest and least often found (5) in group A.

In the evaluation of the changes between the sampling days within each group, significant differences were found in group B for all criteria except the other organ findings, whereas in group A, no significant differences were found, and in group C, only the scores for lung histology and electron microscopy changed significantly over the study period.

## Discussion

### Sequences and phylogenetic analysis

Several reports have shown that ferlaviruses lack species specificity. Ferlaviruses from lizards clustered within groups of snake isolates [11,20,21,24,66] and these squamate ferlaviruses have also been detected in a tortoise with severe respiratory signs [12]. Close genetic relationships between viruses from such different hosts indicate that ferlaviruses may cross species barriers quite readily. These previous observations were also confirmed in the present study, with ferlaviruses isolated from various hosts able to infect and cause disease in corn snakes, although the pathogenicity of the viruses differed markedly.

In this study, the full F and HN genes (CDS region) along with a longer portion of the L gene of 9 ferlavirus isolates were sequenced and analyzed to investigate their taxonomic relationships and to study sequence differences and their impact on viral properties. Earlier publications have characterized many PMV isolates based on sequence analyses of partial L and HN genes [20] or L and F genes [21], and revealed inconsistent clustering into two groups with intermediate isolates [20] or into three groups [21]. This discrepancy was resolved in a later study which suggested revised *sensu lato* A and *sensu lato* B groups [11]. More recent publications have reported the first two representatives of a third PMV cluster: group C, which was equally distant from the other two squamate groups [24,25]. Phylogenetic calculations of the ferlavirus sequences obtained in the present study and those of the currently available homologs from GenBank outlined the same grouping scheme. The SDT analysis of these sequences and its comparison with the similar analysis of sequence counterparts from a closely related genus with more established species and genogroup demarcations helped to prescribe feasible sequence identity demarcation criteria for the ferlaviruses as well. Cut-off values were set to 80 and 90% for the separation of the viral species and the groups, respectively. Thus, we propose that all squamate ferlavirus group (A, B and C) members should be regarded as one viral species, and the tortoise isolate as a separate one.

### Protein motifs, molecular modeling, structural and functional differences

The envelope glycoproteins F and HN have an essential role in viral immunogenicity and pathogenecity and they play multiple roles in virus entry and exit from the host [22,28,29,67,68]. In our analyses, we have found the same domains in the ferlavirus F proteins as described for FDLV [22], and two additional putative heptad repeats (HR3 and -4) in the middle of the F1 part (aa 263–283) and near the C-terminal of the F2 protein section (aa 80–100). This finding was based on *in silico* calculations and models, and also supported by findings in other PMVs [29]. The position of the signal peptide was also calculated differently from that of Franke et al. [22] with a length of 18 or 19 aa in contrast to their 33 aa. The non-conserved aa sequence differences delineating the several groups clustered mostly in this signal peptide and the presumed regulatory region shortly upstream of the furin cleavage site (CS). While the former could not be evaluated, the latter caused a significant conformational change between the 3D models of the different group representatives (Fig 3). Moreover, this structurally divergent small region (aa. 101–105) was detected as a multiple phosphorylation site which shows different patterns in the A, B and C groups. We assume that this newly recognized site might be a fine tuning interface to regulate furin enzyme binding. The formation of this complex interaction may depend on a unique mixture of the steric and electrostatic fit between the furin and the F protein. We found three additional putative phosphorylation sites, located in the cleaved part (F1) of the F protein (Fig 3D). However, the first of these (aa. 136) only becomes accessible for the kinase after furin cleavage. Interestingly, we detected two positions (88 and 360) which significantly influence the electrostatic charge distribution around the furin binding site (Fig 4). We assume that this newly recognized regulating site of the the ferlavirus F protein might be a fine-tuning interface to regulate furin enzyme binding. Consonant with this assumption is that such regulatory sites have also been ascertained for furin recognition in other studies [31,32]. These studies have shown that changes in certain residues of a 20 aa long region around the CL can influence cleavage and, among others, alter viral fusion. Other subtle differences separating the (geno)groups were also found within and around the heptad repeat regions HRA and HRB of the F protein. Single residue changes in these regions have been shown to play a role in the regulation of the 6 helix bundle (6HB) formation, and consequently alter the fusogenic activity of other PMVs, e.g. in simian virus 5 [69] or in Sendai virus [70]. We hypothesize that some or all of the above described differences play a pivotal role in the divergent pathogenicities of the ferlaviruses used in the transmission study.

Paramyxovirus hemagglutinin-neuraminidase (HN) proteins consist of a stalk and globular head, and bind to sialic acid as a receptor. The HN proteins have at least three known functions [71]: (1) with the hemagglutinin activity this protein binds sialic acid receptors at the cell surface. Sialic acid binding sites are mapped to the active sites of the neuraminidase domains in the head of the protein. (2) HN protein has neuraminidase activity. The glycosidic linkages on terminal sialic acid residues are hydrolyzed which leads to destruction of the receptor. (3) HN proteins through direct interactions with the cleaved F protein parts help the metastable F protein to refold into a stable post-fusion form. The fusion activation residues are mapped to the stalk of the HN protein [68]. Inherently, all amino acid variations in this glycoprotein also have an impact on the phylogenetic classification. Even though significant conformational differences were not detected between the three ferlavirus HN proteins used in the transmission study, the newly revealed electrostatic charge pattern differences could easily modify the interaction network of the given HN protein [71].

The transmission studies demonstrated clear differences in pathogenicity between the three virus strains used in the study. This was somewhat expected based on the histories of the original hosts from which the viruses were isolated. However, the glycoprotein analysis indicates that the molecular basis for these marked differences is complex. It is not clear what variation in pathogenicity may be present within the individual genotypes.

### Results of the infection study

The evaluation criteria used in this study ranged from intra vitam clinical assessment to various tissue examination procedures and microbiological examinations, with the tissue being assessed from macroscopic appearence to histologic and finally electron microscopic findings. The techniques and procedures used are in accordance with published references for the assessment of snake lung tissue [13,15,59]. It is obviously a challenge to include all individual findings within a single scoring scheme indicating the severity of the infection. We used a 3-step scoring system, as this allowed a distinct definition of what is to be expected for each score. Only the microbiological results were assessed in a two step score, since any bacteria or fungi isolated from the lung tissue was assessed to be remarkable [18]. This system proved to be practical when used in this study and allowed an overall assessment and comparison of the disease process. However, this overall score is a mere addition of the individual scores and therefore not a direct indicator of the severity of the disease but rather reflects the quanitity of pathological findings. Therefore, no attempt was made to weight the scores for various categories. Beside this quantification of the findings, we further examined the correlations as well as the level of agreement between different criteria to evaluate to what extent the pathological changes correlate with one another, with special attention on correlations between tissue results and the clinical status, the presence of bacteria or fungi and the thickness of the air-blood barrier. These correlations were surprisingly strong, with significant correlations between almost all parameters for all snakes together, and many parameters for the comparison within the groups. For most of these significant correlations, the level of agreement was also significant, demonstrating that the scores between the various criteria matched.

One exception was the comparison with the more unspecific other organ microscopical findings, probably because this category covers a wide range of organ alterations which are of varying importance for the specific disease process. Based on this finding, these organs do not seem to play a specific role in the disease process, although they can be severely affected. The second exception was a lack of significant correlation between the thickness of the air-blood barrier and the presence of bacteria and fungi. This point is discussed later, with respect to the relevance of bacterial isolation. Interestingly, the strongest correlations (Kendall’s tau b = 0.783 to 0.965) were found in group B, which is probably due to the severity of the clinical signs. These correlations demonstrate that with the ongoing disease process, different pathological alterations contribute to the overall clinical condition and lead to clinical disease.

The course of the individual group trials clearly indicated a difference in the virulence of the virus strains used. Beside the interpretation of the individual results within each group and a comparison between the different virus strains used, statistical evaluation was done to calculate if these differences can be confirmed on a significance level, which was, however, not the primary aim of the study.

All examinations in the placebo group revealed normal tissue and unremarkable findings, except for one histologic finding of a mild indication of the infection in the lung tissue. This finding could not be confirmed in the electron microscopy sample. It is conceivable that this was a local reaction to the application of the VH2 supernatant fluid, which was administered without virus load. Since all other diagnostic procedures showed no changes and no pathogen was isolated from the lung tissue, it can be stated that the control group remained healthy.

The virus strain used in infection group A was isolated from a lizard without any clinical signs. The results after application to the corn snakes indicate a successful infection with a moderately virulent strain. In comparison to both other groups, the findings were less dramatic, and no snake died spontaneously or needed to be euthanized until the end of the study. Nevertheless, the consequences of the infection were visible, ranging from macroscopic alterations to electronmicroscopic findings, with 25% of the snakes demonstrating severe histologic alterations, and the tissue findings were significantly correlated to each other (Kendall’s tau b = 0.668). The scoring demonstrated a slow but continuous increase after infection, which also indicates a successful but probably less virulent infection. In this group, it would have been of interest to examine the further development of the disease after the 49-day study period, but this was not possible due to the study design.

Group B was infected with a virus strain isolated after a devastating outbreak in a viper collection, and in comparison to both other groups, the group infected with this virus demonstrated the most severe clinical signs and the transmission study had to be terminated 35 days p.i., earlier than originally scheduled, with a fatal outcome for all remaining snakes. From necropsy on day 2, all snakes demonstrated severe lung alterations, and with one exception (moderate histologic finding in the tissue sample), the histologic and electron microscopic findings were also assessed as severe changes. The scores increased from day 4 dramatically and remained high until the end of the study. The scoring results were strongly correlated, with the highest correlation coefficients in all three groups. These findings indicate that in corn snakes, as in the vipers from which the isolate was originally obtained, this virus was highly virulent and caused a severe and devastating disease.

Corn snakes in Group C were infected with a virus isolated from the same species in a collection with a disease outbreak that caused several sudden deaths. During the trial period three snakes died spontaneously or had to be euthanized, and lung alterations were seen histologically from day 4, while macroscopically all snakes demonstrated severe alterations from day 27, with one exception–one snake was macroscopically and histologically unremarkable at the end of the study, despite positive virus detection and a moderate alteration in electron microscopic assessment. Therefore, this virus strain did not cause severe disease in all snakes.

Comparing the results between the groups statistically, significant differences were found for the lung macroscopy (p = 0.013), the findings in other organs (p ≤ 0.001) and the total scores (p = 0.036). For the first two scores, this significance was then confirmed between groups A and B (P = 0.003), whereas the total score was only significantly different between groups B and C (p = 0.037). This is partly in accordance with the interpretation of the findings as discussed above, as the group B virus strain caused the most severe alterations, whereas the group A strain demonstrated a moderate virulence. The intention for the analyses of this study was descriptive rather than to test an hypothesis postulating significant differences between the virus strains. Therefore, a limited number of animals was used in the individual trials and hence the meaning of the statistical outcome should not be overemphasized.The graphic representation of the total scores (Fig 8) is therefore also relevant for the direct comparison of the course of the disease. Whereas significant differences were found in the gross pathological changes in the lungs, which is a less sensitive method for the detection of changes than others used in this study, no statistically significant differences were found for the much more sensitive method used–the electron microscopic examinations. This can be explained by the fact that alterations on the cellular level occur much earlier than the onset of macroscopic changes and, finally, clinical signs. However, the average scores were different between the groups for these criteria as well, and as expected for the more virulent strain, group B was scored higher than group A. These differences are also reflected in the statistical evaluation of the scores over the sampling days from day 4 to 49: These scores changed significantly in group B for all criteria except the other organ findings, but did not change for any of the parameters in group A. This also indicates a higher impact of the more virulent strain in group B.

The differences in the virulence of the virus strains is also underlined when comparing the lung histology scores over time: group A demonstrated a slow but continuous increase from day 4 to day 49, whereas in group B all snakes were assessed to have severe alterations from day 16 until the end of the study. This demonstrates the relatively rapid pathogenesis of the disease after application of the more virulent virus strain.

The role of bacteria and fungi in the disease process after a ferlavirus infection is unclear, and to fully understand and interpret the bacterial findings, studies on the snake (and reptile) microbiome are necessary. There is some evidence that secondary bacterial infections could have an important influence on the development of pulmonary disease and septicemia [18]. Immunologic responses following ferlavirus infection have been examined with the same material as in this study [37]. It was demonstrated that in group B, the immunologic response to the virus application and lung infection was very weak, which might contribute to secondary bacterial infections, either due to a general weakening of the immune system or due to increased viral replication in the respiratory epithelial cells, and resulting damage and susceptibility to bacteria.

In this study, different Gram negative bacteria were isolated from lung tissue, including six different *Salmonella* serovars, and *Citrobacter freundii* as well as *Klebsiella pneumoniae*. These bacteria can be isolated from the reptile digestive system more or less commonly, but do not belong in the lower respiratory system. Interestingly, in the group with the most severe disease (group B), bacteria were isolated from 58% of the lung tissues tested, whereas in group A (with the lowest scores), bacteria were only detected in 17%. There was a significant correlation between the isolation of bacteria and the lung macroscopy (Kendall’s tau b = 0.512) and histology (Kendall’s tau b = 0.483). This demonstrates the importance of bacterial infections for the development of clinical disease. No correlation was found to the thickness of the air-blood barrier. This might indicate that the air-blood barrier is affected early in the disease process, before bacterial secondary infection leads to more severe lung lesions. The huge spare lung capacity of snakes might explain why these early alterations do not lead to clinical signs of respiratory problems [60].

It is conceivable and supported by the data obtained in this study, that bacterial infections occur–depending on the virus strain, the primary damage to the lung epithelium (and the host immune response [37])–secondary to the viral impact and that these infections have a significant influence on the further disease process, as they contribute to the development of severe lung disease. *Salmonella* spp. seem to play a special role, as has already been suggested [18,64].

## Limitations of the study

The infection part of this study was designed to obtain basic information on the pathology of different ferlavirus strains after experimental infection of a model snake species. Limitations include the relatively low number of samples obtained on each sampling day within the individual infection groups. Further, when interpreting the results, it is important to consider that the experimental infection was done using a high virus load, probably higher than would occur naturally. This might–especially for the time until first clinical signs appear, be of relevance.

The analysis of the glycoprotein structures is based on modeling. Interpretation of correlations between structural differences in the viral glycoproteins and disease pathology in infected snakes is hampered by the complexity of the system, with the immune system and secondary bacterial infections playing a probable role in the observed pathological changes.

For the interpretation of the bacterial isolates and their impact on the development of pneumonia, the range of relevant bacteria might be much broader. Although the infection groups were strictly separated, the animals used for the study were first housed in one room, and neighbouring terraria. Therefore, a collection specific bacterial flora is possible and might have had an influence on the isolates obtained.

## Conclusion

This study demonstrated the devastating impact of different ferlavirus infections on healthy corn snakes. It also showed that ferlavirus strains are able to infect different reptile species, supporting previous findings of genetically similar ferlaviruses in diverse hosts. The established criteria for the definition of individual species within the genus ferlavirus can be used as more genomic information becomes available from ferlaviruses around the world.

The virulence of the ferlavirus infections in this study depended strongly on the virus strain used. The evaluation of the viral glycoproteins demonstrated striking differences between the electrostatic charges of the furin binding sites of the F proteins of the viruses used. These differences are hypothesized to be one of the causes of the differences in virulence between the isolates.

## Supporting information

S1 Table Ferlaviruses analyzed in this study. (DOCX)

S2 Table Primers designed for the RT-PCR amplification of F and HN genes of nine ferlavirus isolates. (DOCX)

S3 Table Primer combinations and cycling conditions used for amplification of HN and F genes of nine *Ferlavirus* isolates. (DOCX)

S4 Table Protein model evaluation statistics. (DOCX)

S1 Fig Alignment of the available ferlavirus complete F proteins, including the novel ones from this report. Consensus sequence and identified or putative domains and motifs of the protein are shown above the alignment. Virus name abreviations are to the left of the sequences (genogroups are indicated in the brackets). For explanation and accession numbers see Fig 1 of the paper. Motif abreviations are as follows: ‘abcdefg’ = heptad repeat unit positions; CL = cleavage site (*sensu stricto* furin recognition site); G = glycosylation site, P = phosphorylation site; RR = regulatory region (extension for the furin recognition site).
(TIF)

S2 Fig Alignment of available ferlavirus complete HN proteins, including the novel ones from this report. Consensus sequence and identified or putative domains and motifs of the protein are shown above the alignment. Virus name abreviations are to the left of the sequences (genogroups are indicated in the brackets). For explanation and accession numbers see Fig 1 of the report. Motif abreviations are as follows: G = glycosylation site, P = phosphorylation site.
(TIF)

S3 Fig Part of the alignment (419–451 aa) of available avulavirus, respirovirus, rubulavirus and ferlavirus complete HN proteins, including the novel ones from this report. Conserved motif “GAEGR” is indicated by orange arrows in the corresponding sequences.
(TIF)
