# Supplementary material for: Three genetically distinct ferlaviruses have varying effects on infected corn snakes (Pantherophis guttatus)
Source: PLoS One. 2019 Jun 4;14(6):e0217164. doi: 10.1371/journal.pone.0217164 (PMC6548425; doi:10.1371/journal.pone.0217164)
Supplement: S1 Table — (DOCX) [file pone.0217164.s001.docx]

| **Isolate name** | **Sub group** | **Host species** | **Host common name** | **References** |
| --- | --- | --- | --- | --- |
| Var GER95 | A | *Varanus prasinus* | Emerald tree  monitor | [4] |
| Xeno USA99 | A | *Xenosaurus platyceps* | Flathead knob scaled lizard | [1,2] |
| Pyth GER01 | B | *Python regius* | Ball python | [2] |
| Crot GER03 | B | *Crotalus horridus* | Timber  rattlesnake | [2] |
| Igu GER00 | B | *Iguana iguana* | Green iguana | [2] |
| Crot GER90 | B | *Crotalus catalinensis* | Santa Catalina Island Rattlesnake | [5] |
| Vip GER90 | B | *Daboia [Vipera] palaestinae* | Palestine viper | [5] |
| Orth GER05 | B | *Elaphe taeniura [Orthriophis taeniurus]* | Beauty snake | [2] |
| Pangut GER09 | C | *Pantherophis guttatus* | Corn snakes | [3] |

References

1. Marschang RE, Donahoe S, Manvell R, Espinal JL. Paramyxovirus and reovirus infections in wild caught Mexican lizards (*Xenosaurus* and *Arbonia* spp.). J Zoo Wildl Med. 2002;33: 317–321.
2. Marschang RE, Papp T, Frost JW. Comparison of paramyxovirus isolates from snakes, lizards and a tortoise. Virus Res. 2009;144: 272–279.
3. Abbas MD, Marschang RE, Schmidt V, Kasper A, Papp T. A unique novel reptilian paramyxovirus: four atadenovirus types and a reovirus identiﬁed in a concurrent infection of a corn snake (*Pantherophis guttatus*) collection in Germany. Vet Microbiol. 2011;150: 70–79.
4. Gravendyck M, Ammermann P, Marschang RE, Kaleta EF. Paramyxoviral and reoviral infections of iguanas on Honduran Islands. J Wildl Dis. 1998;34: 33-38.
5. Blahak S. Untersuchungen zum Vorkommen von Paramyxoviren bei Schlangen und Charakterisierung ausgewählter Isolate. Doctoral thesis, Justus-Liebig-Universität, Gießen, Germany. 1994.
