# Supplementary material for: Three genetically distinct ferlaviruses have varying effects on infected corn snakes (Pantherophis guttatus)
Source: PLoS One. 2019 Jun 4;14(6):e0217164. doi: 10.1371/journal.pone.0217164 (PMC6548425; doi:10.1371/journal.pone.0217164)
Supplement: S2 Table — (DOCX) [file pone.0217164.s002.docx]

| **Primer Name** | **Sequence (5‘→3‘)** | **Position 5’**  **in FDLV** | **Used with isolates of genogroups** |
| --- | --- | --- | --- |
| F gene 130 Fwd | CCRGTCAGYAMWGCRACAATGAT | 5157 | A, B,C |
| F gene 274 Fwd | ACTGAYATYASTGTVVTNGARGG | 5301 | A,B,C |
| F-gene cons 5409 Fwd | GCMCARATMACRGCDGGRATTGC | 5406 | B, C |
| F gene 5755 Rev | GCATTDATRTCRCCATCC | 5735 | B,C |
| F gene 5881 Rev | GGRAARGACATCTCRAT | 5831 | A |
| F-HN cons 6305 Fwd | GAYGGBCTRCARATYAARCC | 6296 | A, B, C |
| F-gene cons 6324 Rev | GGYTTRATYTGYAGVCCRTC | 6316 | B, C |
| F gene 1502 Rev | GCRATAGAGRYHGRAGMDKTYA | 6518 | A, B, C |
| F gene 1631 Rev | AKAAARCCKAGRTTRTTGTAD | 6658 | A, B |
| F-HN cons 7837 Rev | GRGCTTGRCACATRGCYTG | 7829 | A, B, C |
| HN middle 7984 Fwd | TCHCARGTRTGGCTHGGTGCAG | 7974 | A, B, C |
| HN gene 1351 Fwd | GGKAACRRBCCYTGYYCWGC | 8141 | A, B, C |
| HN middle 8335 Rev | TGACTATCWGCMACBCGYCC | 8325 | A, B, C |
| HN cons. 8421 Fwd | GAGYTHAGYCCTGCYACYYTRGG | 8415 | A, B, C |
| HN cons. 9574 Rev | TCATASACYTTATTACCDAG | 9562 | A, B, C |
| L gene 1184 Rev | GARCARAAHACNGCATGYCC | 9910 | A, B, C |
