# Supplementary material for: Three genetically distinct ferlaviruses have varying effects on infected corn snakes (Pantherophis guttatus)
Source: PLoS One. 2019 Jun 4;14(6):e0217164. doi: 10.1371/journal.pone.0217164 (PMC6548425; doi:10.1371/journal.pone.0217164)
Supplement: S3 Table — (DOCX) [file pone.0217164.s003.docx]

| ***PCR Name*** | ***Primer PCR round I***  ***Fwd Rev*** | | ***Primer PCR round II***  ***Fwd Rev*** | | | ***Modification applied to the RT-PCR (Ahne et al., 1999)*** | ***Expected product length (bp)*** |
| --- | --- | --- | --- | --- | --- | --- | --- |
| **F gene I** | 130F | 1631 R | 274F | | 1502R | PCR; 95Cº/45 sec. Annealing;45Cº/45sec. Elongation 72Cº/2min. for both rounds. | 1501,1228 |
| **F gene II** | 257 F | 1631 R | 274F | | 1502R |  | 1374,1228 |
| **F gene III** | 130F | 1631 R | 257F | | 1502R |  | 1501,1245 |
| **F gene VI** | 257 F | 1631 R | 274F | | 1631 R |  | 1374,1357 |
| **F group C** | 257 F | 1631 R | 5409F | | 6324R |  | 1501, 915 |
| **F-HN^±^** | 257 F | HN cons R2∞ | F-HN 6305 | | F 7837R | Annealing; 44 Cº/ 45 sec. Elongation; 72Cº/ 3 min. for both 1^st^ and 2^nd^ rounds. | 2870, 1532 |
| **HN gene** | HN 1F^ǂ^ | HN R2^ǂ^ | HN cons F1∞ | | HN cons R2∞ | RT-PCR has been applied without modifications. | 678, 571 |
| **HN 10 bp^¡^** | 7984 F | 8335 R | 1 round PCR with  45 cycles | | | PCR; 95 C°/ 1 min. Annealing; 45Cº/ 45 sec.Elongation; 72 Cº/ 3 min. | 351 |
| **HN gap^Ɨ^ 400 bp** | HN 1351 F | L 1184 R | 8421F | 9574R | | PCR; 95 C°/ 1 min. Elongation;72 Cº/ 3 min. for both rounds | 1769, 1147 |
| **HN-L** | HN 1303F | L 1217R | HN 1351F | L 1184R | | Annealing; 45Cº/ 45 sec. Elongation; 72Cº/ 3 min. for both rounds. | 1847, 1769 |
