# Supplementary material for: Three genetically distinct ferlaviruses have varying effects on infected corn snakes (Pantherophis guttatus)
Source: PLoS One. 2019 Jun 4;14(6):e0217164. doi: 10.1371/journal.pone.0217164 (PMC6548425; doi:10.1371/journal.pone.0217164)
Supplement: S4 Table — (DOCX) [file pone.0217164.s004.docx]

| **Model name** | **Modelling method** | **Template PDB IDs (**[**https://www.rcsb.org/**](https://www.rcsb.org/)**)** | **Ramachandran (%)**  **(PROCHECK)** | **PROSESS overall quality** | **ProSA**  **Z-score** |
| --- | --- | --- | --- | --- | --- |
| Xeno_F (21-482) | Phyre2^a^ | 5EVM, 2B9B, 1ZTM, 5YZC, 3MAW, 4GIP, 1G5G | 95.9^b^/2.4^c^/1.7^d^ | 3.5 | -7.95 |
| Xeno_F (21-482) | I-TASSER | 5EVM, 2B9B | 94.7/3.9/1.4 | 2.5 | -9.63 |
| Crot_F (21-482) | Phyre2 | 5EVM, 2B9B, 1ZTM, 5YZC, 3MAW, 4GIP, 1G5G | 96.8/2.2/1.0 | 3.5 | -8.14 |
| Crot_F (21-482) | I-TASSER | 5EVM, 2B9B | 94.5/3.9/1.6 | 2.5 | -8.44 |
| PanGut_F (21-482) | Phyre2 | 5EVM, 2B9B, 1ZTM, 5YZC, 3MAW, 4GIP, 1G5G | 95.6/2.7/1.7 | 3.5 | -8.25 |
| PanGut_F (21-482) | I-TASSER | 5EVM, 2B9B | 95.9/3.2/0.9 | 2.5 | -9.06 |
|  |  |  |  |  |  |
| Xeno_HN (86-564) | Phyre2 | 4JF7, 1V3E, 4FZH, 1USR, 1Z4X, 5B2C, 4UF7, 5NOP, 3D1, 2RKC, 3ALX | 93.1/4.1/2.8 | 2.5 | -6.91 |
| Xeno_HN (86-564) | I-TASSER | 1V3E, 1V2I, 4MZA, 4UF7 | 93.1/3.1/3.8 | 2.5 | -6.94 |
| Crot_HN (86-564) | Phyre2 | 4JF7, 1V3E, 4FZH, 1USR, 1Z4X, 5B2C, 4UF7, 5NOP, 3D12, 2RKC, 3ALX | 97.6/1.4/1.0 | 5.5 | -7.04 |
| Crot_HN (86-564) | I-TASSER | 5B2C, 1Z4X | 92.4/4.4/3.2 | 3.5 | -6.85 |
| PanGut_HN (86-564) | Phyre2 | 1V3E, 4JF7, 4FZH, 1USR, 1Z4X, 5B2C, 4UF7, 5NOP, 3D12, 2RKC, 3ALX | 96.4/2.2/1.4 | 2.5 | -8.25 |
| PanGut_HN (86-564) | I-TASSER | 1Z4X, 5B2C | 93.4/4.1/2.5 | 2.5 | -9.06 |

^a^ Only those template PDB IDs are indicted in this table which have 100% confidence value in the Phyre2 output summary.

^b^ Allowed regions

^c^ Generously allowed regions

^d^ Disallowed regions
