# Supplementary figures and images for: Three genetically distinct ferlaviruses have varying effects on infected corn snakes (Pantherophis guttatus)
Source: PLoS One. 2019 Jun 4;14(6):e0217164. doi: 10.1371/journal.pone.0217164 (PMC6548425; doi:10.1371/journal.pone.0217164)

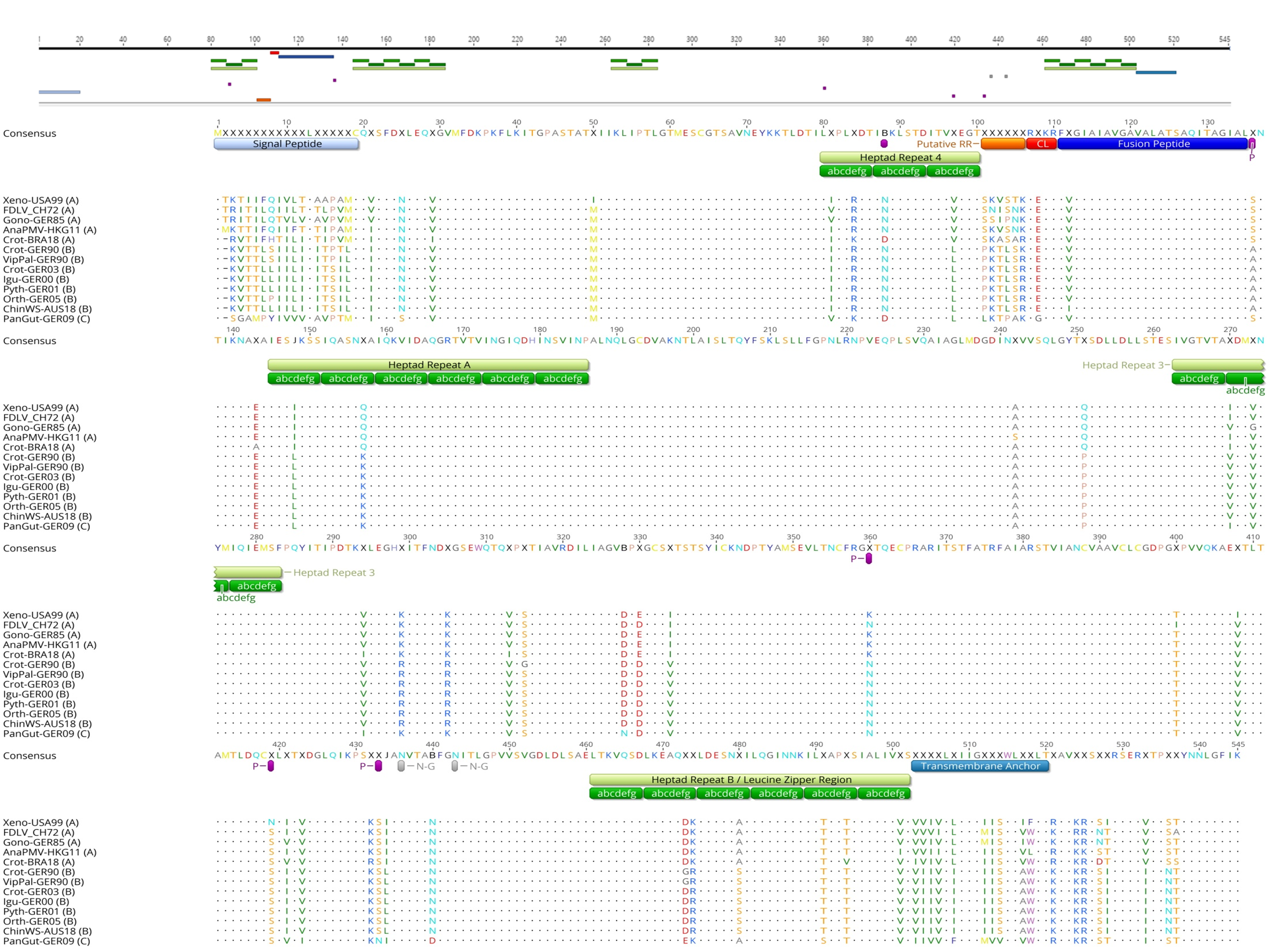

Supplement: S1 Fig — Consensus sequence and identified or putative domains and motifs of the protein are shown above the alignment. Virus name abreviations are to the left of the sequences (genogroups are indicated in the brackets). For explanation and accession numbers see Fig 1 of the paper. Motif abreviations are as follows: ‘abcdefg’ = heptad repeat unit positions; CL = cleavage site (sensu stricto furin recognition site); G = glycosylation site, P = phosphorylation site; RR = regulatory region (extension for the furin recognition site). (TIF) [file pone.0217164.s005.tif]

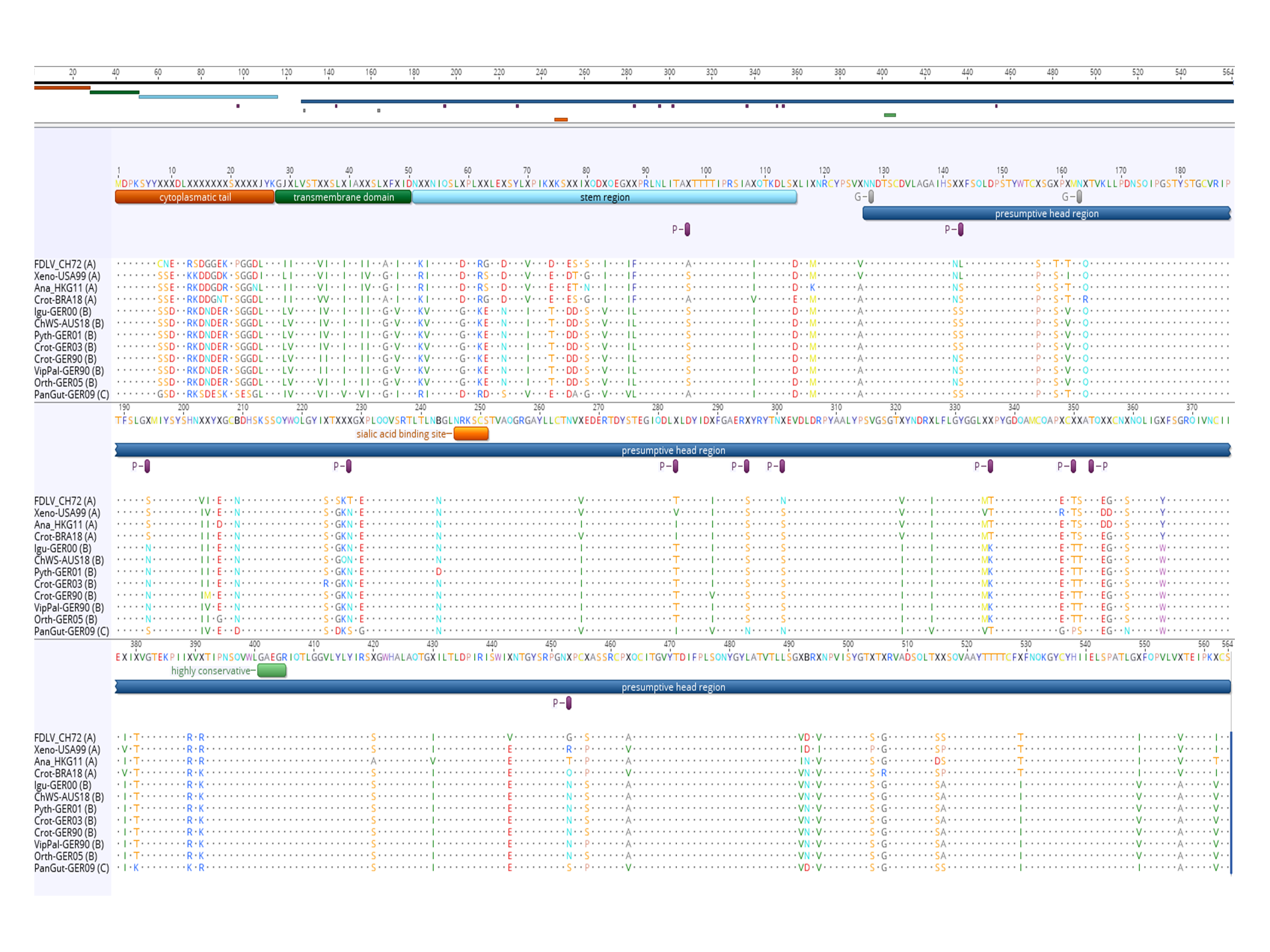

Supplement: S2 Fig — Consensus sequence and identified or putative domains and motifs of the protein are shown above the alignment. Virus name abreviations are to the left of the sequences (genogroups are indicated in the brackets). For explanation and accession numbers see Fig 1 of the report. Motif abreviations are as follows: G = glycosylation site, P = phosphorylation site. (TIF) [file pone.0217164.s006.tif]

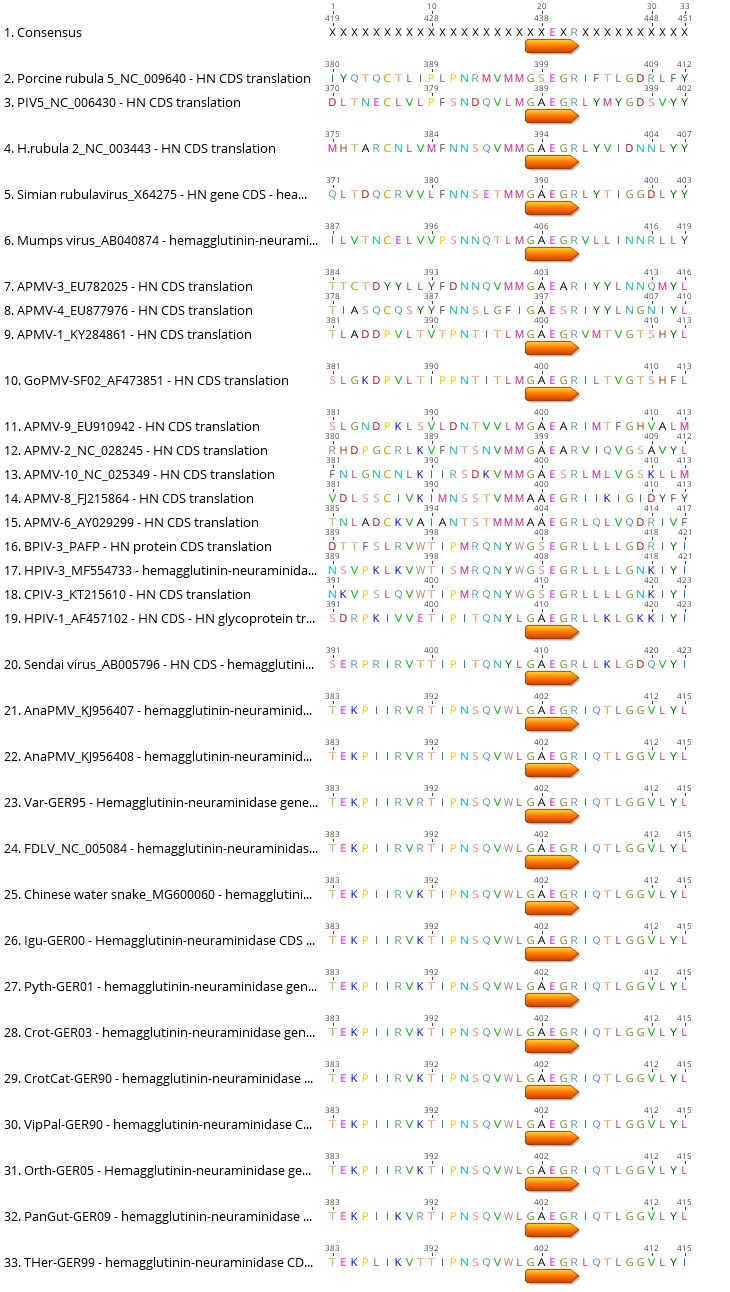

Supplement: S3 Fig — Conserved motif “GAEGR” is indicated by orange arrows in the corresponding sequences. (TIF) [file pone.0217164.s007.tif]
